# Pareto optimal metabolic engineering for the growth‐coupled overproduction of sustainable chemicals

**DOI:** 10.1002/bit.28103

**Published:** 2022-04-21

**Authors:** Matteo N. Amaradio, Varun Ojha, Giorgio Jansen, Massimo Gulisano, Jole Costanza, Giuseppe Nicosia

**Affiliations:** ^1^ Department of Biomedical & Biotechnological Sciences University of Catania Catania Italy; ^2^ Department of Computer Science University of Reading Reading UK; ^3^ Department of Biochemistry University of Cambridge Cambridge UK; ^4^ Department of Drug Science University of Catania Catania Italy; ^5^ National Institute of Molecular Genetics Milan Italy

**Keywords:** Pareto optimal metabolic engineering, genome‐scale metabolic models, *Y. lipolytica*, β‐carotene production, *S. cerevisiae*, succinate production

## Abstract

Our research aims to help industrial biotechnology develop a sustainable economy using green technology based on microorganisms and synthetic biology through two case studies that improve metabolic capacity in yeast models *Yarrowia lipolytica* (*Y. lipolytica*) and *Saccharomyces cerevisiae* (*S. cerevisiae*). We aim to increase the production capacity of beta‐carotene (β‐carotene) and succinic acid, which are among the highest market demands due to their versatile use in numerous consumer products. We performed simulations to identify in silico ranking of strains based on multiple objectives: the growth rate of yeast microorganisms, the number of used chromosomes, and the production capability of β‐carotene (for *Y. lipolytica*) and succinate (for *S. cerevisiae*). Our multiobjective optimization methodology identified notable gene deletions by searching a vast solution space to highlight near‐optimal strains on Pareto Fronts, balancing the above‐cited three objectives. Moreover, preserving the metabolic constraints and the essential genes, this study produced robust results: seven significant strains of *Y. lipolytica* and seven strains of *S. cerevisiae*. We examined gene knockout to study the function of genes and pathways. In fact, by studying the frequently silenced genes, we found that when the *GPH1* gene is knocked out in *S. cerevisiae*, the isocitrate lyase enzyme is activated, which converts the isocitrate into succinate. Our goals are to simplify and facilitate the in vitro processes. Hence, we present strains with the least possible number of knockout genes and solutions in which the genes are turned off on the same chromosome. Therefore, we present results where the constraints mentioned above are met, like the strains where only two genes are switched off and other strains where half of the knockout genes are on the same chromosome. This study offers solutions for developing an efficient in vitro mutagenesis for microorganisms and demonstrates the efficiency of multiobjective optimization in automatizing metabolic engineering processes.

## Graphical abstract

Pareto Optimal Metabolic Engineering Framework. A multiobjective evolutionary algorithm takes a population of “*m*” strains (genetic vector on the far left) and simultaneously optimizes yield and growth rate that are computed using flux balance analysis. The framework produces a Pareto Front (on the far right), from which this study selects seven Pareto optimal strains of *Yarrowia lipolytica* and seven strains of *Saccharomyces cerevisiae*. These strains are Pareto Optimal strains with the least knockout genes, maximal yield and growth rate and genes silenced on the same chromosome(s). Such strains simplify and facilitate the production of sustainable chemicals.

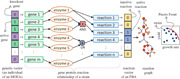

## 1 INTRODUCTION

Synthetic biology tools offer a cost‐efficient and eco‐compatible alternative to the traditional high energy rate manufacturing processes for producing renewable feedstocks (King, Dräger et al., 2015; King, Lloyd, et al., 2015; Nielsen & Keasling, 2016). In this field, mathematical modeling can help find optimal genetic manipulations (Stracquadanio et al., 2010) and speed up the identification of better‐performing strains to produce specific metabolites of interest (Umeton et al., 2011) that can replace their petrochemical‐derived equivalents. The yeast models are the bioreactors of metabolic precursors, which are converted into a wide range of consumer products, for example, beta‐carotene (β‐carotene) and succinic acid (or succinate).

The β‐carotene has nutraceutical and antioxidant properties. These properties make β‐carotene a highly desired product in agriculture, food, pharmaceutical, and industries alike. In 2018, it had an estimated $1.4 billion in market demand (Abdel‐Mawgoud et al., 2018; Larroude et al., 2018). Furthermore, the high versatility of *succinic acid* in multiple industrial chemical applications and consumer products makes its market demand grow steadily at a compound annual growth rate of around 27.4% to reach $1.8 billion (768 million metric tons (MT) at $2.3 kg^−1^) by 2025 (Nghiem et al., 2017).

The β‐carotene and succinic acid are mainly produced by specific host yeasts: the *oleaginous yeast Yarrowia lipolytica* (*Y. lipolytica*) is the preferred host to produce carotenoids (β‐carotene) because of its naturally high supply of carotenoids precursor cytosolic as acetyl‐CoA and redox cofactor: nicotinamide adenine dinucleotide phosphate (*NADPH*), which *reduces nicotinamide adenine dinucleotide* (Kildegaard et al., 2017). On the other hand, the preferred succinic acid producer yeast is *Saccharomyces cerevisiae* (*S. cerevisiae*), known for its ability to grow under acidic conditions and for its well‐characterized role in wine acidity (Cao et al., 2013; Franco‐Duarte et al., 2017; Vilela, 2019).

Our study proposes a robust methodology based on a multiobjective evolutionary algorithm (MOEA) for in silico identification of competitive genetically manipulated strains of *Y. lipolytica* and *S. cerevisiae* metabolism (Section 2). This is through selecting effective gene deletions (knockout) in the respective genome‐scale metabolic (GEM) models. In addition, the algorithm attempts to knock out genes on the same chromosome to simplify the in vitro process. We describe the results of the two case studies in Section 3. The results show that our methodology is an effective strategy to optimize the design of competitive strains to produce organic compounds in large‐scale fermentation that leads to a more targeted in vitro mutagenesis (King, Dräger et al., 2015; King, Lloyd, et al., 2015; Nielsen & Keasling, 2016; Patanè et al., 2015, 2019).

The β‐carotene is the main source of provitamin A; this is its main nutritional function. Vitamin A is also known as retinol, which is used as a visual pigment chromophore in the eyes. It is also implicated in the growth and reproductive efficiency of the epithelial tissue. Thus, retinoids have been used in dermatological treatments, such as for acne. The antioxidant property of carotenoids is linked to their capacity to bind with singlet oxygen by a conjugated double bond system. Moreover, β‐carotene is used as a food colorant and as a nutritional supplement of vitamin A. In fact, 100% of β‐carotene can be converted into vitamin A. The international trade of β‐carotene is dominated by private companies such as Roche and BASF.

These companies produced β‐carotene through a synthetic approach. Both companies started the synthesis using the same molecule, namely, β‐ionone, but used different methods. Roche used a synthesis for polyenic aldehydes in the form of enol–ether condensation. BASF used the Witting condensation for the production of β‐carotene. The aspect that enhances the interest in the natural β‐carotene is that it contains several other carotenoids in low concentrations, which provides further health benefits. Only a low percentage of the total β‐carotene produced worldwide is natural, and it is really interesting to increase the natural production of this chemical and make the process more environmentally friendly (Ribeiro et al., 2011). It was illustrated that globally β‐carotene production via herbal sources comprises 2%. Carotenoids are isolated from components of flowers, plants, and fruits. They can be found in vegetables and in fruits, in which orange carrots are the most common source of β‐carotene. As said above, industrial carotenoids are produced by extraction and chemical synthesis; chemical synthesis produces hazardous wastes, which are harmful to the environment (Gupta et al., 2022).

The succinic acid is traditionally made from fossil resources used in different ways, such as a chemical intermediate in medicine, in the manufacture of lacquers, and perfume. An interesting application field is that it can be used as an intermediate for producing biodegradable polymers (Rex et al., 2017). In fact, succinic acid was selected as one of the top bio‐based chemicals. Moreover, it can be converted into other valuable chemicals such as 1,4‐butanediol and tetrahydrofuran. In 2015 the total market for succinic acid was hovering around 30,000–50,000 tons per year, but this value has grown, probably because the knowledge of technology for bio‐based production has increased. Four companies (Reverdia, Succinity, Bioamber, and Myriant) commercialized the processes for the bio‐based production of succinic acid. Also, it works on the conversion of succinic acid to various derivatives such as polybutylene succinate by BioAmber, Sinopec. The versatility of this molecule has allowed the development of an increasing interest in the synthesis, extraction, and purification of this chemical, with processes that will increase the yield and especially make the process more environmentally friendly (Choi et al., 2016).

## 2 IN SILICO ENGINEERED STRAIN AND CASE STUDIES FOR YEASTS

### 2.1 Automated in silico strain design of genome‐scale models

The genome‐scale models are among the most effective tools for in silico representation and analyses to tackle strain design and optimization tasks (King, Dräger et al., 2015; King, Lloyd, et al., 2015; Lu et al., 2019; Nielsen & Keasling, 2016; Palsson, 2015). Such genome‐scale models can include a *metabolic network* of pathways or organisms that offers a representation of microorganisms as realistic as possible (Palsson, 2015). The metabolic network reconstruction is based on the *stoichiometry* of metabolic chemical reactions and metabolites involved as products or reagents.

In the stoichiometry of metabolic chemical reactions and metabolites, the stoichiometric coefficients are grouped in a sparse matrix S of size m×n, wherem is the number of metabolites in the model (each row is a unique compound) andn is the number of simulated reactions (each column is a reaction). The matrix S permits the definition of a system ofm mathematical constraints, given by a mass balance equation: Sv=0, wherev is a vector of variables representing flux through all reactions in a steady‐state. Solving Sv=0 is known as flux balance analysis (FBA) (Palsson, 2015).

The constraints  Sv=0 ensure that the balance of mass for each metabolite in a model holds. Moreover, in all reconstructions, m<n, due to more reactions than the metabolites). However, such a system of constraints defines an *unconstrained* solution space. Therefore, a *feasible solution space* is usually defined by applying a lower bound and upper bound$lb_{i}$ and$ub_{i}$ on each flux$v_{i}$. These bounds are applied to internal reactions and external exchange reactions simulating the uptake and secretions of chemicals to and from the extracellular regions. These bounds mainly represent the environmental conditions in which a cell is located and its metabolic footprinting.

Once a feasible solution space is defined, it is possible to select a specific pointv that is optimal for a specific objective function $v_{\mathrm{bio}}$, which is defined as linear combination vectorsv andc, wherec is a vector of weights indicating the quantity of each reaction (flux intensity) that contributes to the objective function. The objective function usually maximizes flux through a single artificial reaction simulating biomass production or the *growth rate*. The definition of this objective function is crucial for the precision of fluxes. This stoichiometric definition of objective is often complicated in both its composition and values of coefficients, even though it only refers to the exponential growth phase of a cell's cycle. The optimal point v can be found by optimizing systems by solving mmol gDW^−1^ h^−1^  and by using linear programming as

(1) $$\mathrm{maximize}\;\sum_{j}^{n}c_{j}v_{j\;}=c^{T}v=v_{\mathrm{bio}},\mathrm{subjected}\;\mathrm{to}\;\mathrm{Sv}=0\;\text{and}\;lb_{i}\leq v_{i}\leq ub_{i},\;\mathrm{for}\;\mathrm{all}\;i=1,\ldots ,n.$$

When the optimal valuev of the growth rate is established, a subsequent optimization called flux variability analysis (FVA) (Palsson, 2015) is performed to explore a more *constrained feasible solutions space* where this new constraint is defined by hyperedges of the polytope. FVA allows the definition of flux ranges for a single reaction in the hyperedge.

The range (lower and upper bounds) is critical for evaluating the robustness of single strain prediction. For example, if we consider the production of a compound, a small range could mean that, despite the variance that the predictions might have along the edge‐link in the reaction graph, there is still a minimum production always predicted regardless of other fluxes, and this prediction is comparable to the theoretical maximum under stress growth conditions. The parsimonious FBA (pFBA) is another approach that could lead to more reliable prediction, specifically for the fluxes of internal metabolic reactions occurring in the internal compartments, different from the extracellular region (Palsson, 2015).

Similar to FVA, the pFBA approach calculates the optimal growth rate from FBA optimization as a constraint and then minimizes the sum of absolute values of fluxes through all reactions in a network. Thus, this new optimization returns a parsimonious distribution of fluxes (optimal array of fluxes) throughout the network, avoiding numerical predictions that do not have any biological justification, a common issue in FBA (Palsson, 2015). For an optimal array of fluxes, we define two quantities to evaluate the results of chemical production: theyield andproductivity of specific chemicals. These quantities are, respectively, defined as:

(3) $$\mathrm{yield}=\frac{v_{\mathrm{bio}}}{\;v_{\mathrm{cs}}}\;$$

and

(4) $$\mathrm{productivity}=h^{-1}=\frac{v_{\mathrm{bio}}\;}{v_{\mathrm{cs}}}\cdot v_{\mathrm{obj}}.$$

After normalizing yield ≤ 1 using the corresponding molar masses $v_{\mathrm{cs}}$, the yield becomes a scalar quantity, expressing the fraction of chemicals produced over the quantity of glucose $v_{\mathrm{glc}}$ (in general, the quantity of carbon source $v_{\mathrm{cs}}$) that the cell has used. The theoretical upper bound for yield equals 1, that is, when all the glucose is converted to the specific chemical without waste. The productivity, instead, is measured as (h^−1^), which gives the rate of speed at which a product can be obtained, where $v_{\mathrm{obj}}$ is the growth rate. Moreover, Using the extreme ranges obtained from the FVA for each reaction, the ranges for yield and productivity can be obtained, and this will give us the energy and carbon source quantity that each reaction uses.

The predictions on the distribution of reaction fluxes obtained in wild‐type (WT) and *simulated conditions* come from evaluating the metabolic network. The next question is then how the linear programming problem must be changed to reproduce the metabolic engineering techniques, namely, in our case, to simulate the gene deletions. We use an MOEA to address this issue.

### 2.2 Pareto optimal metabolic engineering framework

**Figure 1:**
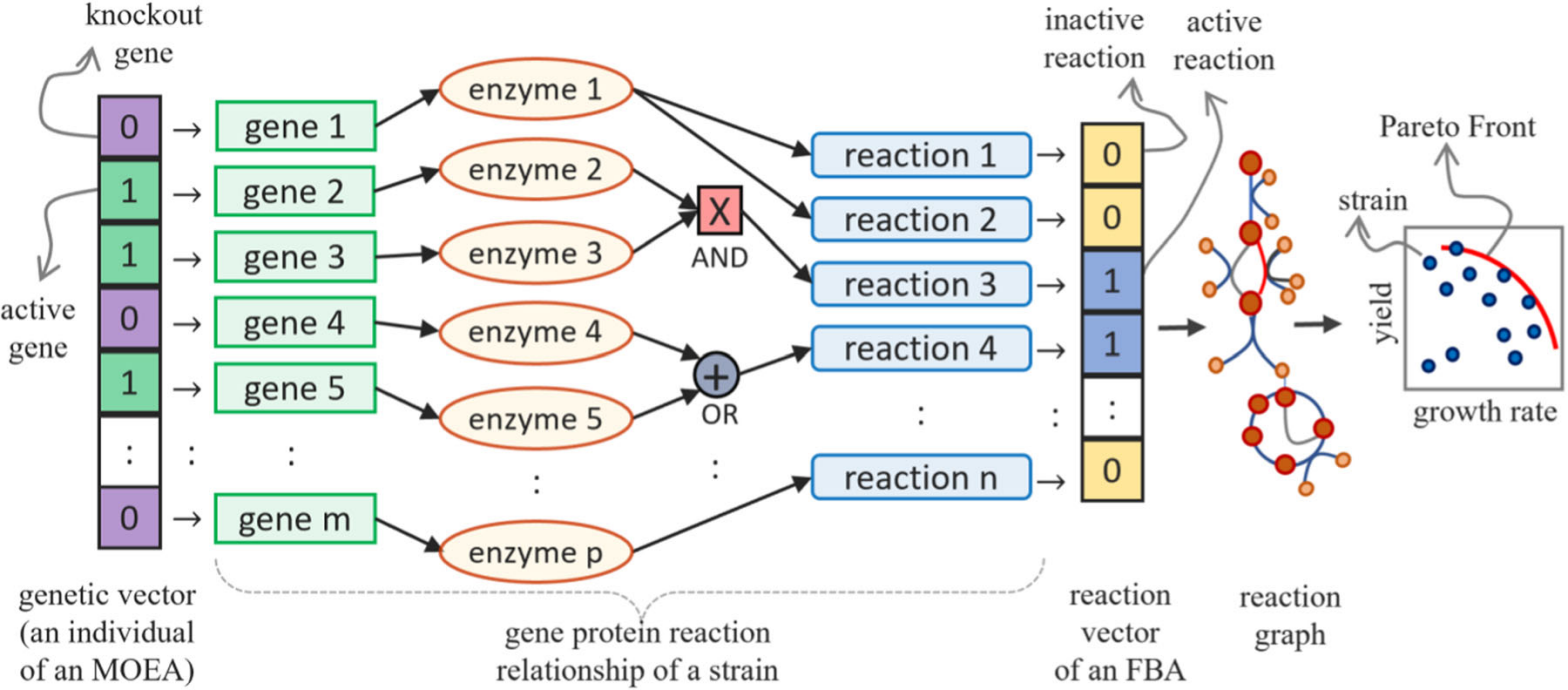
Metabolic engineering frameworks. This framework takes a multiobjective evolutionary optimization algorithm (MOEA) of a population of “*m*” individuals (genetic vector of genes on the far left in the framework) for optimizing yield and growth rate. This optimization produces a Pareto Front (on the far right in the framework) computed using flux balance analysis (FBA). The FBA takes a reaction vector of length “*n*” formed by a combination of “*p*” enzymes. Active genes in the genetic vector are indicated with 1, and the active reactions (flux), created based on genes and enzyme rules (“X” indicates AND “+” indicates OR), in FBA are indicated with 1 in the reaction vector. Value 0 in the genetic vector indicates knockout genes, and 0 in the reaction vector indicates inactive reaction.

We created our metabolic engineering framework (Figure 1) by applying MOEA on genome‐scale models of yeasts, where the fitness functions of the models were computed using FBA. As explained in Figure 1, the genes are present in the genome‐scale models through gene protein reaction (GPR) relationships that link the genes with the coded enzymes and coded enzymes with the corresponding catalyzed chemical reaction.

Using a series of logical rules defined by atomic propositions referring to single genes of the simulated genome and logical connectors AND OR, the genes are simulated in the model as a prerequisite for each reaction to be active. The logical connectors help represent complex relationships, such as the isoenzymes and subunits. The rules for each reaction can be evaluated from the atomic true and false values, and the reactions with satisfied propositions (that refer to genes) are considered in the metabolic network. A reaction that must be excluded from the model following one or more gene deletions is simulated, posing the lower and upper bounds equal to 0 and forcing the corresponding flux variable to assume a null value.

Multiobjective optimization algorithms are used for this class of problems since an exhaustive evaluation of all the possible gene deletion combinations is practically infeasible, even using a simple approach such as FBA. The number of different configurations to be considered for such a study is equal to $2^{g}$ for g genes. Even when considering a fixed maximum number of deletions, it would be equal to a sum of ∑d(g) for d gene deletions (knockout). Hence, a multiobjective optimization framework is useful for efficiently exploring the large solution space of gene deletions. We propose using a modified version of the multiobjective metabolic engineering algorithm (Patané et al., 2019) to explore the possible sets of deletions and their impact on the growth rate and chemical production (see Supporting Information in Section A). It is an ad hoc evolutionary algorithm that follows the principles of natural selection (De Jong, 2016). That means it starts from an initial population of candidate strains (logical arrays in which each value represents a gene), and the population evolves over a fixed maximum number of generations. In each generation, genetic operators such as mutation and crossover attempt to switch genes on and off (i.e., randomly switch binary components of the array). Then, the selection operator selects the candidates with the most promising and best‐performing value of one or more *fitness functions* (*objectives*). The fitness functions, in our case, are *growth rate* and *production capacity*. Such an iterative process evolves a population in the next generation fitter than the previous generation and helps obtain the near‐optimal solutions in the final populations.

The optimization of two or more objectives, which in most cases compete for resources available for the cell from the exchange reactions, defines a classical *multiobjective optimization problem*, in which it is possible to define a set of optimal points, called *Pareto Front* (Patanè et al., 2015). Each point in a Front cannot be improved in all the considered objective functions simultaneously. An optimization technique for these problems aims to find such a Front or a good approximation of the problem. The code of this framework is available at: https://github.com/GiuseppeNicosia1/pareto-optimal-metabolic-engineering.

## 3 RESULTS

### 3.1 β‐Carotene production in engineered *Y. lipolytica*

#### 3.1.1 Optimal strains of β‐carotene production by *Y. lipolytica*

The production of β‐carotene in *Y. lipolytica* is simulated and evaluated using the iYL619_PCP genome‐scale model (Pan & Hua, 2012). In addition, based on our literature review, to promote β‐carotene production in *Y. lipolytica*, we added the heterologous metabolic pathway of three knock‐in genes: geranylgeranyl diphosphate synthase (*GGS1* from *Y. lipolytica*), phytoene synthase/lycopene cyclase, and phytoene dehydrogenase (*carPR* and *carB* from *Mucor circinelloides*) (Celińska et al., 2017; Gao et al., 2014; Larroude et al., 2018). Consequently, the model was modified by adding the corresponding arch (a new pathway) to the metabolic network. After introducing three knock‐in genes, the next step is to maximize the expression of these genes within the yeast. This can be implemented by engineering the yeast or by biotechnology strategies like Celińska et al. (2017), where they found that β‐carotene production was enhanced by increasing lipogenesis and gene copy number and by identifying the best combination of promoters and genes. For this, they performed a promoter shuffling strategy by using a golden gate toolbox for *Y. lipolytica*.

There are several other examples of the strategy to optimize *Y. lipolytica*. For example, Zhang et al. (2020) enhanced the β‐carotene production by increasing copies of carB (three copies) and carRP (two copies) genes and overexpressing the genes (GGS1, ERG13, and HMG) correlated with Mevalonate pathway. This pathway contributes to the production of carotenoid precursors: isopentenyl pyrophosphate and dimethylallyl pyrophosphate (DMAPP). The DMAPP metabolite is converted into farnesyl diphosphate (FPP) in multiple‐step reactions. The overexpression of the GGS1 enzyme makes the whole of FPP convert into geranylgeranyl pyrophosphate (GGPP) rather than entering into the squalene pathway. From GGPP, two knock‐in genes carRP and carB convert GGPP into phytoene and lycopene, and in the final step of the pathway, carRP converts lycopene into β‐carotene. Moreover, Zhang et al. (2020) successfully analyzed 11 sites for CRISPR/Cas9‐mediated heterologous gene knock‐in *Y. lipolytica* and found that four sites are involved in β‐oxidation (POX2, POX3, POX4, and POX6), six sites belonged to nonfunctional pseudogenes due to frameshift (E1, A1, B1, A2, F1, and E2), and the last site LIP1 is engaged in lipid metabolism.

Liu et al. (2021) suggested a modern strategy to optimize the β‐carotene production by constructing codon‐adapted genes and minimizing the intermediate accumulation, which plays an important role in metabolic balance. The metabolic balance means no accumulation of intermediates at the connecting node when combining upstream and downstream pathways. This methodology inserts the β‐carotene biosynthesis pathway consisting of knock‐in genes carRA and carB from *Blakeslea trispora*, where these two genes were codon adapted for a better expression. Here, metabolic balance is an important factor. In the biosynthesis of β‐carotene, there are four enzymes that limit the rate of the process: tHMGR, GGS1, carRA, and carB. The metabolites (intermediates) that are converted by these enzymes are HMG‐CoA, FPP, GGPP, lycopene, and phytoene. Therefore, an inadequate expression of these enzymes will lead to a high accumulation of these intermediates, which results in a small production of β‐carotene. Hence, they overexpressed the genes tHmgR, Ggs1, carRA, and CarB with Snf, Lip1, Pox3, and Pox4 as the target sites, which caused the deletion of these genes that led to increases in lipid body formation that allowed more storage space for β‐carotene.

F. Yang et al. (2021) proposed a new approach that focuses on a new feature called *DID2* genes. This gene is a subunit of the ESCRT (endosomal sorting complex required for transport). This complex is made up of cytosolic protein complexes known as ESCRT‐0, ESCRT‐I, ESCRT‐II, and ESCRT‐III that, together with other accessory proteins, enable a remarkable way of membrane remodeling. The *DID2* gene in *Y. lipolytica* was amplified and inserted into pJN44, leading to pJN44‐Did2. As a subunit of the ESCRT protein complex, the *DID2* improves β‐carotene production (increased by 260%) and does not cause metabolic stress for the host cell. To understand why this gene improved β‐carotene production in *Y. lipolytica*, they studied the messenger RNA (mRNA), protein, and precursor that are part of the β‐carotene pathway. From the study of mRNA of *Thmg*, *Ggs1*, *carRA*, and *carB* genes, they realized that due to the introduction of the *DID2* gene, the mRNA levels of β‐carotene pathway genes were high. In their study, *DID2* elevated the mRNA level of the β‐carotene synthesis pathway genes in *Y. lipolytica*. They found that the *DID2* also increased glucose consumption during the exponential growth phase and stationary phase, which is an important feature in metabolic engineering.

**Figure 2:**
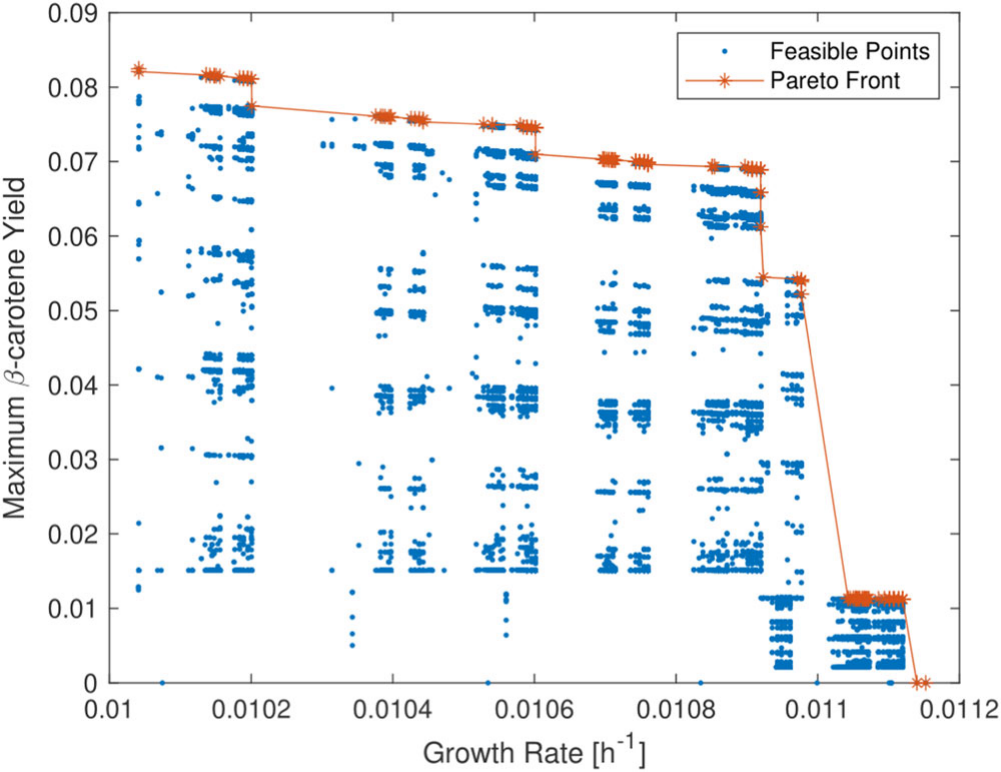
Pareto Front (red asterisk and connected with red line) obtained by a multiobjective evolutionary algorithm for optimizing growth rate (*x*‐axis) and yield (*y*‐axis) of β‐carotene in *Y. lipolytica*. The phenotypes cluster feasible points in several regions related to specific gene deletions (blue points). Each cluster characteristically changes the prediction of growth rate (h^−1^) and β‐carotene *yield* based on specific gene knockout.

In our study, for an in silico simulation, we set the growth conditions of yeast and configured two main objectives: maximization of β‐carotene production and maintenance of biomass as close as possible to that of the WT. We employ MOEA for retrieving gene deletions leading to suboptimal strains. MOEA starts from a population of WT strains with the highest growth rate prediction, but a null β‐carotene production and is located at the bottom of the Pareto Front (see Figure 2). Then, MOEA explores possible deletions and phenotypes of the resulting strains. The phenotypes tend to cluster in various separate regions. We discovered that these regions are related to specific gene deletions that characteristically change the predictions. Hence, the Fronts in Figure 2 appear to be divided into steps. However, the clusters vary only slightly from each other. The MOEA finds the importance of a small set of genes that share a similarity in predictions through its evolutionary optimization procedure. The presence of these clusters highlights the algorithm's efforts in finding other useful deletions. This is particularly evident in the top region of Figure 2, where the algorithm considered several points. Still, none led to other Pareto optimal points, despite the sensible reduction in the growth rate.

Similarly, we analyzed the productivity of the yeast. The productivity values are in the order of magnitude of −4 because both growth rate and production have low values. Furthermore, the glucose level varies among the points, especially those with low growth where the metabolic network does not use all the carbon sources. These differences also change the distribution of the explored points and the Pareto Front; many points have various productivity levels that do not correspond to the productions. Notably, not all the points of the Pareto Front (in Figure 2) are optimal when considering either productivity or growth rate. Instead, there is a clear trade‐off. Thus, the best way of comparing the strains can vary depending on the desired phenotypes and the specificity of applications.

**Figure 3:**
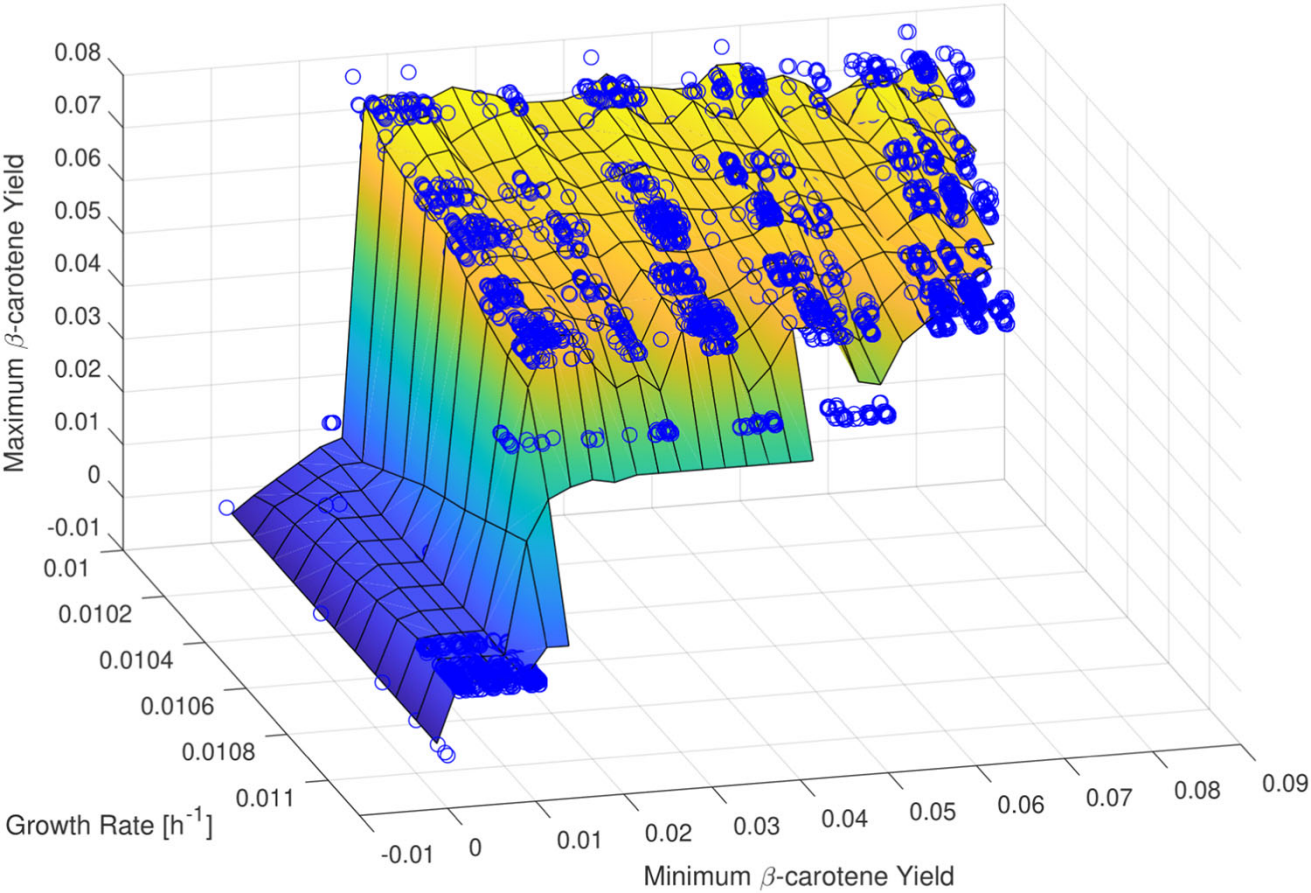
Characterization of each strain for their minimum (*x*‐axis) and maximum (*z*‐axis) β‐carotene yields against the corresponding growth rate (*y*‐axis). The most significant strains are located at the top right corner of the graph, as they have high values for growth rate (e.g., 0.011) and maximum β‐carotene yield (e.g., 0.08) and minimum β‐carotene yield (0.08).

Figure 3 represents a three‐dimensional graph to compare minimum productivity, maximum productivity, and growth rates to determine the characteristics of strains. These three parameters are essential to determine which strains are best suited for use in the laboratory. The parameters maximum and minimum productivity measure the quantity of β‐carotene production under optimal and nonoptimal conditions. Indeed, it is necessary to find a high value of the maximum and minimum productivity (see *x*‐axis and *z*‐axis in Figure 3) to achieve high production of β‐carotene. Finding a high value of minimum productivity is more significant because it is challenging to maintain stable optimal growth conditions in both in vitro and industrial bioreactors. Hence, a strain where the value of minimum productivity is high ensures a high β‐carotene production is possible even in suboptimal conditions. This gives us a measure of how resistant an obtained strain is. In Figure 3, the most significant strains are at the top right of the graph because they represent the best compromise between yeast growth and β‐carotene productivity.

#### 3.1.2 Carbon source analysis

The relationship between the production of the chemical and the amount of used carbon source measures the yield. For example, our study used glucose as a carbon source for yeast growth and to produce β‐carotene. However, different carbon sources can also be used, for example, glycerol (GLY).

Larroude et al. (2018) used GLY as a *cheap carbon source* with two different media, rich yeast extract peptone dextrose (YPD)medium and synthetic yeast nitrogen base (YNB) medium, and with different carbon source concentrations (10, 20, 30, and 60 g L^−1^), keeping the amount of nitrogen constant. They selected culture media YPD10, YPD60, YNB20, YNB30, YNB60, and YNBGLY60 for β‐carotene production and found that the production varied significantly based on culture media usage. Additionally, Braunwald et al. (2013) showed that both the carbon–nitrogen ratio and the applied initial carbon and nitrogen contents influenced the parameters, that is, high carbon–nitrogen ratio promotes lipid production and other carbon‐based molecules such as carotenoids. However, they found that lipid yield was not affected by ammonium contents, while the carotenoid production decreased significantly at low and high ammonium supply levels.

Therefore, we can suggest that carbon source has little influence on production since glucose and GLY have similar titer and yields. In addition, a clear correlation between the increase in the initial glucose content and the production titer was found in both rich and synthetic media (Larroude et al., 2018). Noticeably, the production yields for all the YNB‐based media were similar and were independent of the amount or kind of carbon source used. However, this was not the case for rich media (YPD), and it offered a trade‐off between production titer and yield. For example, the best β‐carotene titer was 1.5 g L^−1^ in YPD60, while the best yield was 0.048 g g^−1^ in YPD10. Moreover, in all cases, YPD had higher titers and yields than YNB. (Larroude et al., 2018). Therefore, they selected rich media to further optimize the culture conditions in a controlled fermentation in a bioreactor.

#### 3.1.3 Analysis of strains and relative genes knockout

We analyzed the results of the MOEA simulation to identify the most suitable strains for in vitro testing. Table 1 offers growth values of β‐carotene production, ATP, NADH, NADPH, and especially FAD(H2). From Table 1, we identified that strain number 7 (row 7 in Table 1) has a significant exponential increase in its energy value parameters, that is, the values of ATP, NADH, NADPH, and FADH have notably increased (see columns in Table 1). Specifically, the production of ATP rises by 108.72%, NADH by 116.60%, NADPH by +146.05%, and FAD(H2) by +603.74% for strain number 7. This exponential growth can bring either positive or negative results, which means that a significant amount of energy is required to grow yeasts, but such a substantial concentration of molecules can produce toxic substances as well.

**Table 1:** The seven significant strains of *Yarrowia lipolytica* selected from the Pareto Front.

No. of strain	Biomass (WT variation)	β‐carotene production (mmol gDW^−1^ h^−1^)	ATP production (WT var. %) (mmol gDW^−1^ h^−1^)	NAD(H) production (WT var. %) (mmol gDW^−1^ h^−1^)	NADP(H) production (WT var. %) (mmol gDW^−1^ h^−1^)	FAD(H2) production (WT var. %) (mmol gDW^−1^ h^−1^)	No. of KO
WT	0.011152362	0	62.68911709	11.83086467	29.84459341	0.128944987	
1	**0.01112 (−0.29%)**	0.031725	62.7378 (+0.08%)	11.5504 (0.19)	29.7883 (−0.19%)	0.12895 (0%)	1
2	0.010907 (−2.20%)	0.22634	73.8947 (+17.87%)	14.7743 (+24.88%)	32.5591 (+9.10%)	0.90737 (+603.69%)	3
3	0.010897 (−2.29%)	0.22736	69.2036 (+10.39%)	14.7437 (+27.40%)	32.5388 (+9.03%)	**0.91146 (+606.86%)**	4
4	0.010897 (−2.29%)	0.22736	69.2036 (+10.39%)	14.7437 (+27.40%)	32.5388 (+9.03%)	**0.91146 (+606.86%)**	4
5	0.010885 (−2.40%)	0.22736	73.8978 (+17.88%)	14.776 (+24.89%)	32.5414 (+9.04%)	**0.91146 (+606.86%)**	5
6	0.010838 (−2.82%)	**0.22737**	73.7797 (+17.69%)	14.7484 (+24.66%)	32.4141 (+8.61%)	**0.91146 (+606.86%)**	6
7	0.010619 (−4.78%)	0.22637	**130.8433 (+108.72%)**	**25.6254 (+116.60%)**	**73.4325 (+146.05%)**	0.90744 (+603.74%)	6

*Note*: We selected seve strains based on the parameters shown in columns. The first row shows results on wild‐type (WT) strain. Column 1 is the numbering of strains. The second column is the biomass (the total mass of all living material in a specific area, habitat, or region). Other columns from left to right are β‐carotene production (the quantity of β‐carotene produced by strains), adenosine triphosphate (ATP) production, NAD(H) production, NADP(H) production, FAD(H2) production. The last column is the number of knockout genes of strains. We focused primarily on two parameters: biomass and β‐carotene production. Thus, we selected the strains with smaller biomass loss and higher β‐carotene production. The highest values are indicated in bold.

**Figure 4:**
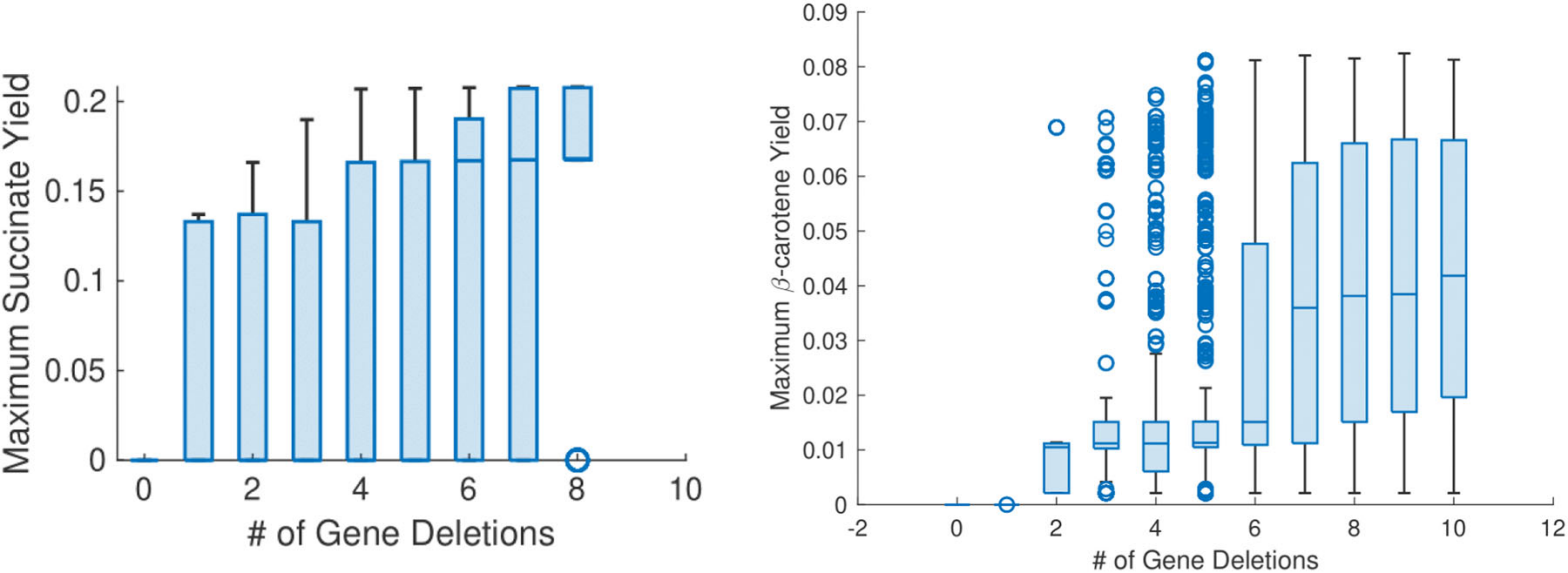
Correlation between yield and the number of gene deletions. The *x*‐axis shows the number of knockout genes in ascending order. Yield along the *y*‐axis is the ratio between the production of chemicals and the quantity of consumed carbon source. The mean yield is shown by a horizontal blue line within the box plot. Left plot: Correlation between maximum β‐carotene yield and the number of gene deletions (knockout genes) in strains obtained from *Yarrowia lipolytica*. Right plot: Correlation between maximum succinate yield and knockout genes in *Saccharomyces cerevisiae*.

Additionally, Table 1 shows that the strains for the β‐carotene production fluctuate only marginally. This is due to the metabolic pathway obtained by adding three knock‐in genes, which were common to all the strains, and they only differ in their knockout genes. Table 2 identifies the number and type of genes removed (knocked out) from the genome of the selected strains. We observed that there were between 1 and 7 genes switched off. Hence, to analyze knockout genes and the involved pathway, we used the Kyoto Encyclopedia of Genes and Genomes (Kanehisa & Goto, 2000). Moreover, we studied the relationship between yield and the number of knockout genes. This relation is shown in Figure 4 (left plot), which indicates that the increase in yield is directly proportional to the number of knockouts.

**Table 2:** Knockout (KO) genes of *Yarrowia lipolytica*

No. of strain	No. of KO	Gene KO
1	1	YALI0F17996g
2	3	YALI0A05379g, YALI0F11935g, YALI0F17996g
3	4	YALI0A5379g, YALI0E22649g, YALI0F15587g, YALI0F17996g
4	4	YALI0A5379g, YALI0B15598g, YALI0F15587g, YALI0F17996g
5	5	YALI0A04983g, YALI0A05379g, YALI0E22649g, YALI0F15587g, YALI0F17996g
6	6	YALI0A04983g, YALI0A05379g, YALI0C23408g, YALI0E22649g, YALI0F15587g, YALI0F17996g
7	6	YALI0A05379g, YALI0C04433g, YALI0D06325g, YALI0E16643g, YALI0E26004g, YALI0F17996g

*Note*: Results identify the genes that were removed from the genome of *Y. lipolytica* of each strain and explain this removal. Strains in rows are arranged in the ascending order of the number of their KO genes. The frequently occurring KO genes are YALI0A05379g and YALI0F17996g.

Table 2 allows biotechnological considerations to be made. Namely, as mentioned above, the following is given: the number of genes deleted in each strain, the name of each gene, and the chromosomal location of each. The chromosome belonging to each gene is clarified by the name. Specifically, the chromosome is indicated in the sixth character of the identifier of the corresponding gene. Knowledge of the associated chromosome becomes useful in the transition from in silico to in vitro. This is because the deletion of genes belonging to the same chromosome is greatly facilitated by knockouts.

We examined the strains from Table 2, in which most of the knockout genes were located on the same chromosome. For example, in strain number 2, we notice three knockout genes (YALI0A05379g, YALI0F11935g, and YALI0F17996g); as we can see from the name, the first gene is located on chromosome A and the other two on chromosome F. In this strain, we have a β‐carotene production of 0.22634 (mmol gDW^−1^ h^−1^). We further study the frequently silenced genes *YALI0F17996g* and *YALI0A05379g* in Table 2. The gene *YALI0F17996g* has a length of 4527 bp, GC% = 53.15%, and is a part of chromosome F. This gene translates to a protein that catalyzes the reaction of ergosterol transport. The gene *YALI0A05379g* has a length of 2361 bp, GC% = 51.16%, and is part of chromosome A. This gene translates an enzyme, chorismate:l‐glutamine aminotransferase, for *para*‐aminobenzoate (PabA) synthase ABZ1. This enzyme is composed of two parts, PabA and PabB. In the absence of PabA and glutamine, PabB converts ammonia and chorismate into 4‐amino‐4‐deoxychorismate (in the presence of Mg^2+^). On the other hand, the PabA converts glutamine into glutamate only in the presence of stoichiometric amounts of PabB. Additionally, this enzyme is coupled with EC 4.1.3.38, aminodeoxychorismate lyase, to form 4‐aminobenzoate. Thus, the reaction catalyzed by this enzyme is chorismate + l‐glutamine → 4‐amino‐4‐deoxychorismate + l‐glutamate, and the standard Gibbs free energy (Δ*rG*′°) for this reaction is −2.0558853 kcal mol^−1^, which is an exergonic reaction. Therefore, this indicates a spontaneous reaction.

To establish the role of *YALI0F17996g* and *YALI0A05379g*, we set up an additional simulation, where we knocked in the heterologous genes (*GGS1*, *carPR*, and *carB*) and knocked out *YALI0F17996g* and *YALI0A05379g*. We obtained an increase in β‐carotene productivity and a lower d‐glucose exchange than a WT strain (see Table 3).

**Table 3:** Comparison between wild type (WT) and strains obtained by deletion of YALI0A5379g and YALI0f17996g genes, which were frequently silenced during multiobjective optimization.

Reactions	WT	Deletion of YALI0A5379g and YALI0f17996g
Growth rate (h^−1^) (WT variation %)	0.011152362	0.010919286 (−2.16%)
Max productivity (h^−1^)	0	0.062331504
Min productivity (h^−1^)	0	0.059657794
d‐glucose exchange (WT variation %) (mmol gDW^−1^ h^−1^)	−3.034654555	−3.631218038 (−16.42%)

### 3.2 Succinic acid production in engineered *S. cerevisiae*

#### 3.2.1 Optimal strains of succinate production by *S. cerevisiae*

We changed the MOEA framework to tackle critical points that could affect the precision and reliability of the results for our case study on succinic acid production in *S. cerevisiae*. The analysis of the succinic acid production in *S. cerevisiae* is conducted using the GEM yeast model, v. 8.3.1 (Lu et al., 2019). In this genome‐scale model, no changes were needed as the pathway for succinate production is already included in the model. Furthermore, for these simulations, we set the bounds of the external exchange reactions of the models to simulate the growth in a rich medium, that is, the synthetic defined medium for the *S. cerevisiae* model.

First, we improved the framework based on the knowledge obtained from β‐carotene production. We found that some obtained points offered a low growth rate, which signifies an impeded cell metabolism. Therefore, we limit the tolerance of the MOEA to a reduced growth rate compared to the WT. We set a bound of 10% on this growth rate.

Second, using the fluxes predicted by the pFBA, we induced a similar bound on the sum of the fluxes through the reactions in the network that produce the metabolites ATP, GTP, NADH, NADPH, and FADH2. These constraints aimed to improve the quality of the results obtained by the algorithm, ensuring that the strains do not differ excessively from the WT at every step. In other words, we force the algorithm to explore more extensively a narrow region to produce better results.

**Figure 5:**
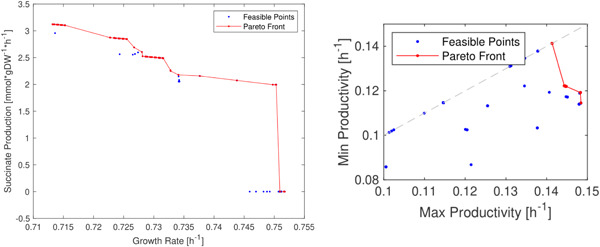
Succinic acid production in *Saccharomyces cerevisiae*. Pareto Fronts are shown in red and feasible solutions are in blue dots. Left plot: The trade‐off between the competitive objectives (succinate production vs. growth rate) constitutes the observed Pareto Front. Right plot: The trade‐off between the competitive objectives (min and max productivity) constitutes the observed Pareto Front. Minimum productivity is computed to highlight the most robust strains.

Third, we included some restrictions on the gene deletions by not allowing MOEA to delete some of the essential genes and allowing the deletions of genes involved in the synthetic double deletions. For this, we use the database of Heavner and Price (2015). This restriction helped the algorithm avoid unfeasible mutated strains that are not always correctly predicted by FBA (Heavner & Price, 2015). Finally, we considered the maximization of productivity. The results of the algorithm using this setting are summarized in Figure 5 (left plot), where we observe a slight reduction in allowable growth. Nonetheless, the algorithm increased the productivity from the initial null point. Thus, a step‐like clustering behavior is still present, but less prominent than the one obtained for β‐carotene shown in Figure 2.

Contrary to the previous simulation, Figure 5 (left plot), instead of growth rate, our final simulation in Figure 5 (right) included two extremes: *min* and *max* productivity values as per FVA. The resulting strains of this simulation are shown in Figure 5 (right). The number of points in this simulation is in only half of the phenotypic space. The points lying on the highlighted line (dashed line in Figure 5 (right)) correspond to strains for which the range of productivity collides with a single value. We speculate that these strains are more robust as the in silico productivity is always ensured when a higher value of min productivity is obtained.

For example, the only point of the Pareto Front on the dashed line in Figure 5 (right plot) is a strain with five gene deletions. Compared to the other points of the front, the points (strains on the dashed line) have lower maximum productivity, but have the highest minimum productivity predicted. Thus, the points (strains) with the highest min productivity values should be preferable to the other points to ensure a less competitive minimum prediction, even though other parameters such as the metabolic footprinting should also be considered along with min productivity.

#### 3.2.2 Analysis of strain in succinate production by *S. cerevisiae*

The analysis of the knockout genes through FBA answers the questions: how knockout of a specific gene influences the production of a specific metabolite within a cell and how it changes the metabolism of yeast. Mathematically, this process is described by a GPR map (Orth et al., 2010). In GPR, the organism's genes are grouped using “Boolean” relationships, which associate each gene to a group based on common reactions catalyzed by their respective associated proteins. From the analysis of *S. cerevisiae*, we obtained 483 strains derived from gene deletions, which resulted from exploring possible sites of gene deletion by MOEA.

MOEA results provided important information about the choice of strains for in vitro testing and the actual succinate production. For example, Figure 4 (right plot) shows the relationship between the maximum succinate yield changes and the number of knockout genes. Here, we observe that the value of succinate yield increases when the number of knockout increases. This is obvious because the silencing of genes spares energy that strains might use to produce succinate.

For our research, we used a set of parameters to select strains. First, we select seven significant strains (shown in Table 4). After this selection, we focused on analyzing genes. Mainly, we focus on identifying knockout genes of each strain and their position and length within the genome of *S. cerevisiae*. In addition, between three and nine genes were silenced on average for each strain, as shown in Table 4. Table 5 reports silenced genes of each strain, and a roman number in the third column in Table 5 explicitly locates chromosomes to a silenced gene it belonged to.

**Table 4:** The seven significant strains of *Saccharomyces cerevisiae* selected from the observed Pareto Front.

Max productivity (h^−1^)	Min productivity (h^−1^)	Max yield	Min yield	Succinate production (mmol gDW^−1^ h^−1^)	Biomass (WT variation)	KO
0	0	0	0	0	0.751751629	WT
0.14064	0.10696	**0.2395**	0.19708	2.9562	0.71361 (−5.07%)	4
0.14795	0.114	0.2295	**0.20685**	3.1027	**0.71528 (−4.85%)**	4
0.14795	0.114	0.2295	**0.20685**	3.1027	**0.71528 (−4.85%)**	4
0.14795	0.114	0.2295	**0.20685**	3.1027	**0.71528 (−4.85%)**	4
0.14795	0.114	0.2295	**0.20685**	3.1027	**0.71528 (−4.85%)**	4
0.14795	0.114	0.2295	**0.20685**	3.1027	**0.71528 (−4.85%)**	4
**0.14841**	**0.11455**	0.2282	0.2081	**3.1215**	0.71319 (−5.13%)	9

*Note*: The parameters from left to right are max productivity, min productivity, max yield, min yield, succinate production, biomass, and knockout (KO) genes. The values of parameters succinate production and biomass are used to select seven strains. The strains in rows are arranged in the ascending order of the number of their KO genes. Row 1 indicates wild‐type (WT) strain. The highest values are indicated in bold.

**Table 5:** Knockout (KO) genes of *Saccharomyces cerevisiae*.

KO	Gene KO	Gene KO (standard name) and chromosomic locations
4	YDL171C, YPL061W, YPR160W, YPR127W	GLT1 (IV), ALD6 (XVI), GPH1 (XVI), YPR127W
4	YBR221C, YDL171C, YPL061W, YPR160W	PDB1 (II), GLT1 (IV), ALD6 (XVI), GPH1 (XVI)
4	YDL171C, YGR193C, YPL061W, YPR160W	GLT1 (IV), PDX1 (VIII), ALD6 (XVI), GPH1 (XVI)
4	YDL171C, YNL071W, YPL061W, YPR160W	GLT1 (IV), LAT1 (XIV), ALD6 (XVI), GPH1 (XVI)
4	YDL171C, YER178W, YPL061W, YPR160W	GLT1 (IV), PDA1 (V), ALD6 (XVI), GPH1 (XVI)
4	YDL171C, YHR002W, YPL061W, YPR160W	GLT1 (IV), LEU5 (VIII), ALD6 (XVI), GPH1 (XVI)
9	YDL171C, YHR144C, YJR105W, YLR209C, YNL071W, YNL169C, YOR175C, YPL061W, YPR160W	GLT1 (IV), DCD1 (XII), ADO1 (X), PNP1 (XII), LAT1 (XIV), PSD1(XIV), ALE1 (XV), ALD6 (XVI), GPH1 (XVI)

*Note*: For each strain in rows, the number of silenced (KO) genes are reported in column 1, names of KO genes are reported in column 2, and the chromosomes they belong to are indicated in column 3 by a roman number (note that the budding yeast *S. cerevisiae* has a 16‐chromosome organization). In column 2, from left to right, the acronym provides a description of the genes, where Y indicates yeast's unknown sequence, the second letter represents the chromosome, the third letter indicates the left or right arm of the chromosome, the number indicates the sequence of the open‐reading frame (ORF), and the last letter W or C represents Watson (5′ → 3′) or Crick strand, respectively. Column 3 shows that the standard name of a gene is composed of three letters followed by a number and a roman number written in brackets indicating the chromosome it belongs to, the final letter if an uppercase character indicates a dominant gene, while if it is a lowercase character, it indicates a recessive gene.

MOEA, importantly, knockout a varied number of genes for strains, leading to heterogeneous results. For example, Table 4 shows that up to nine genes were knocked out, and despite a large number of knockouts, the algorithm was able to simulate the life of yeast and, crucially, had an increased succinate production. Thus, intuitively, knockout genes result from an optimal compromise between the cost of knockout and the production rate of succinate. The analysis of genes was carried out through Genemania (Warde‐Farley et al., 2010) software for predicting functions and involved pathways. Similar to our analysis of knockout genes in β‐carotene, we characterize the genes that were silenced with higher frequency in succinate production. Table 5 identifies frequently silenced (knockout) genes of *S. cerevisiae*. These are *GLT1*, *ALD6*, and *GPH1*.

The knockout gene *GLT1* of *S. cerevisiae* encodes for a glutamate synthase (GOGAT), which is essential in central nitrogen metabolism (CNM). CNM contains two pathways (glutaminases [GDA] and GOGAT, which is NADH‐dependent, converts one molecule of glutamine and one molecule of α‐ketoglutarate into two glutamate molecules; Guillamon et al., 2001) for glutamate biosynthesis using glutamine as the sole source of nitrogen. The presence of two pathways makes it harder to choose the most significant routes for the biosynthesis of the end product.

Although the pathway GDA (glutaminases)‐encoding genes are unknown, these glutaminases may exist because mutants grow well on glutamine even without the GOGAT enzyme. Some authors (e.g., Tempest et al., 1970) have suggested that the role of the GOGAT pathway, with the concerted action of the glutamine synthetase, is to assimilate ammonium and synthesize glutamate even under a shortage of ammonium. However, NADPH‐dependent glutamate dehydrogenase (NADPH‐GDH) is used to incorporate ammonia during a shortage or excess of nitrogen in other microorganisms. This hypothesis suggests that NADPH‐GDH is the main pathway for glutamate biosynthesis. Therefore, physiological studies have reported that in GOGAT or NADPH‐GDH activities, both WT and mutant strains are impaired. These show that GOGAT has different roles in different microorganisms (Barel & MacDonald, 1993; Valenzuela et al., 1998), but its function in *S. cerevisiae* is still unclear. Although the clear reason for *GLT1* knockout could not be established, the unclear role of GOAGT in *S. cerevisiae* may conclude that *GLT1* may have less influence on succinate production and the growth of the yeast.

The ALD6 gene of *S. cerevisiae* encodes the cytosolic Mg^2+^‐activated NADP‐dependent acetaldehyde dehydrogenase (ACDH) and exhibits 60% and 30% activity of wild‐type activated ACDH (Remize et al., 2000). The main cytosolic Mg^2+^‐activated ACDH isoform preferentially uses NADP, and this isoform plays an important role in both ethanol (deletion of ALD6 gene disables the organism to use ethanol as a carbon source) and glucose. Since the deletion of a mutant is feasible on glucose, the enzyme encoded by *ALD6* is not solely responsible for producing cytosolic acetyl‐CoA (Meaden et al., 1997) and thus not solely responsible for succinate production.

The *GPH1* gene of *S. cerevisiae* translates to glycogen phosphorylase enzyme, which is an essential allosteric enzyme in carbohydrate metabolism, but not essential for the life of the yeast. Hence, this gene draws our attention because we need to answer our question: “why does the genetic algorithm knock out this gene frequently?” To do this, using Escher (King, Dräger et al., 2015 King, Lloyd, et al., 2015), we analyzed the strains in which we knocked out one by one all of these genes. In the case of the *GPH1* gene, we noticed a particular rearrangement of the metabolism. We found that the yeast's response to gene knockout was to activate the transcription of the isocitrate lyase enzyme. This enzyme catalyzes the conversion of isocitrate into succinate. Therefore, we found evidence that the MOEA algorithm can find new information for the production of specific chemicals (see Supporting Information in Section C).

We also analyzed the second strain of Table 4, in which four genes have been knocked out (PDB1, GLT1, ALD6, and GPH1), with a model that includes Expression and Thermodynamics FLux (ETFL), which efficiently integrates RNA and protein synthesis with traditional GEM models. To adapt this model for *S. cerevisiae*, Oftadeh et al. (2021) developed yETFL, in which they increase the original formulation with supplementary considerations for biomass composition, the compartmentalized cellular expression system, and the energetic costs of biological processes. The results of this analysis are that the strain with four knockout genes loses 1.52% of the growth rate compared to the WT.

## 4 CONCLUSIONS

We developed an automated tool for in silico implementation of genetic deletions and a precision modulation of the phenotype of the host yeasts to select optimized strains. We implemented an MOEA and its refinements to optimize strains to obtain results that can realize a sustainable synthesis of metabolic precursors used in large‐scale manufacturing processes. Our approach optimizes two yeasts *Y. lipolytica* and *S. cerevisiae*. This provides many strains with varied features for selecting the best strains based on the production capacity of chemicals (β‐carotene and succinate) with the lowest biomass losses. By examining knockout genes, we characterize pathways influenced by the knockout. We found seven strains of *Y. lipolytica* and seven strains of *S. cerevisiae* capable of producing a high amount of β‐carotene and succinate. Such in silico processes of strains creation save costs, leading to a high biosustainability.

## Supporting information

Supplementary information.
