# Supplementary material for: Pareto optimal metabolic engineering for the growth‐coupled overproduction of sustainable chemicals
Source: Biotechnol Bioeng. 2022 Apr 21;119(7):1890–902. doi: 10.1002/bit.28103 (PMC9321710; doi:10.1002/bit.28103)
Supplement: Supplementary file 1 — Supplementary information. [file BIT-119-1890-s001.pdf]

Supplementary Information

Pareto Optimal Metabolic Engineering for the Growth-coupled Overproduction of Sustainable Chemicals

Matteo N. Amaradio[1,*], Varun Ojha[2,*], Giorgio Jansen[3,*], Massimo Gulisano[4], Jole Costanza[5], Giuseppe Nicosia[1,3]

[1]Department of Biomedical & Biotechnological Sciences, University of Catania, Catania Italy
[2]Department of Computer Science, University of Reading, Reading, United Kingdom
[3]Department of Biochemistry, University of Cambridge, Cambridge, United Kingdom
[4]Department of Drug Science, University of Catania, Catania, Italy
[5]National Institute of Molecular Genetics, Milan, Italy

Summary:

This supplementary Section is divided into six sections. Section A describes the 'pseudocode' used in our work. Section B presents a comparison table of different strategies used for the production of carotenoids, where we specifically focus on β-*Carotene production.* Subsequently, Section C compares different studies based on succinate production and different strategies used for its fabrication. Section D describes the analyses conducted by silencing the three genes (GLT1, ALD6, and GPH1) of interest for S. cerevisiae. Finally, Section E supplements a list of abbreviations.

A. Pseudocode

The pseudocode presented in Algorithm 1 is based on the NSGA-II algorithm (Deb 2001). The NSGA-II algorithm works by sampling from the optimization problem input domain an initial set of candidate solutions to the optimization problem, i.e., a population, and it iteratively attempts to optimize the problem objective function by applying to the population a set of *evolutionary operators* (Deb 2001). The parameters of the algorithm are (1) *pop,* the size of the population; (2) *maxGen,* the maximum number of *generations* to be performed; (3) *dup,* the strength of the *cloning operator* (Ciccazzo et al. 2008); and (4) *uKC,* the maximum knockout cost allowed to be taken into account by the algorithm (Patanè et al. 2019). Therefore, we have an initial population that is randomly initialized by *Initpop,* which samples the domain of the problem by applying a few random mutations to the wild-type strain. Then, we apply FBA to each strain in the population of candidate strain $P^{(gen)}$ in order to evaluate the production rate of metabolites and the corresponding growth rate; we measure rank and crowding distance for each member of the population. Rank ensures the Pareto-orientation of our procedure, redirecting the search towards the problem Pareto Front. The *crowding distance is* a rough estimation of the population density near each candidate solution.

During the main loop of the optimization, candidate solutions in *unexplored* regions of the objective space (thus having small values of crowding distance) are preferred to those which lie in "crowded" regions of the objective space. This has the purpose of obtaining good approximations of the actual Pareto Front of the problem. We then initialize the generation counter and enter the main loop, which is performed *maxGen* times. At the beginning of each generation, the *Selection* procedure generates a mating pool $Pool^{(gen)}$ by selecting individuals from the current population $P^{(gen)}$. This is done following a *binary tournament selection* approach. Namely, tournaments are performed until there are [*pop*/2] individuals (*parents*) in the mating pool. Each tournament consists of randomly choosing two individuals from $P^{(gen)}$ and putting the best of the two individuals (in terms of rank and crowding distance) into $Pool^{(gen)}$. *Children are* thus generated from the *parents* by using binary *mutation*. Namely, we randomly generate *dup* different children from each parent, generating the $Q_{dup}{}^{(gen)}$. Then, we keep only the best solution of these *dup* children for each parent, hence defining the actual offspring set $Q^{(gen)}$. This is because many of the mutations allowed in an FBA model are *lethal* mutations, i.e., they severely compromise the bacteria's growth. Of course, a greater value for *dup* implies that feasible mutations are more likely to be found, whereas smaller values reduce the computational burden of the optimization. In order to achieve this, we first ensure that each individual of $Q^{(gen)}$ is feasible with respect *dup* to our optimization problem (i.e., it has less than *uKC* knockouts). Namely, if a child is not in the allowed region, we randomly knockin genes until it is forced back to the feasible region.

We, therefore, evaluate the biomass and metabolites production of each new individual, and the algorithm computes new values of rank and crowding distance for each individual. Procedure *BestOutOfDup* selects

from each of the *dup* children of each parent the best one and puts it in the $Q^{(gen)}$ set. Finally, procedure *Best* generates a new population of *pop* individuals, considering the current best individuals and children. The output of the optimization algorithm is the union of the populations of all the generations. We then analyze the optimization results by means of Pareto analysis, hence computing the *observed* Pareto fronts, i.e., the set of $\cup_{gen} P^{(gen)}$ elements which are not dominated by any other element in $\cup_{gen} P^{(gen)}$ (Notice that $\cup_{gen} P^{(gen)}$ covers only a portion of the feasible region. Hence, we talk about observed Pareto optimality).

Algorithm 1. Pareto Optimal Metabolic Engineering (POME) Algorithm

POME Algorithm (*Genome-scale metabolic model, medium, carbon source,* Oxygen level, *pop*, *maxGen*, *dup*, *uKC*)

objective_function_1 ← Biomass /* maximization of the biomass */

objective_function_2 ← β-Carotene production (or Succinate Production, Yield and Productivity) /* maximization of the chemical or maximization of the Yield/Productivity */

objective_function_3 ← Number_of_Used_Chromosomes /* Minimization of the used chromosomes */

gen ← 0

$P^{(gen)}$ ← *InitPop*(*pop*)

FBA($P^{(gen)}$)

Rank_and_crowding_distance($P^{(gen)}$)

while (*gen* < *maxGen)* **do**

   $Pool^{(gen)}$ ← *Selection*($P^{(gen)}$,[*pop/2]*)

   $Q_{dup}^{(gen)}$ ← *GenOffspring*($Pool^{(gen)}$, *dup*)

   $Q_{dup}^{(gen)}$ ← *Force_to_feasible*($Q^{(gen)}$,*uKC*)

   FBA($Q_{dup}^{(gen)}$)

   Rank_and_crowding_distance($Q_{dup}^{(gen)}$)

   ($Q^{(gen)}$) ← *BestOutOfDup*($Q_{dup}^{(gen)}$, *dup*)

   $P^{(gen+1)}$ ← *Best*($P^{(gen)} \cup Q^{(gen)}$, *pop*)

   gen ← *gen* + 1

end_while

return ($\cup_{gen} P^{(gen)}$)

B. Comparison of algorithms and methods for β-Carotene production

In this section, we present a list where we integrate information about our work (we added our best strains) and other research to provide an exhaustive summary of methods and strategies used to produce β-Carotene (see Table B). We debated not only Y. lipolytica but other microorganisms: *Blakeslea trispora, Escherichia coli, Saccharomyces cerevisiae, Corynebacterium glutamicum, Rhodobacter sphaeroides, and Xanthophyllomyces dendrorhous.* The engineering strategies utilized are different. For example, in the case of *Blakeslea trispora,* Mantzouridou et al. (2005) used the control of Oxygen transfer rate (OTR). This methodology scales the OTR under steady-state conditions where the dissolved oxygen concentration remains constant. Under this condition, the OUR (Oxygen uptake rate) of the microorganism equals the OTR. The OUR is calculated by means of an oxygen-mass balance around the reactor, and since OUR = OTR, OTR is quantified as a result. An oxygen-mass balance around the reactor is conceptually defined as: Rate of oxygen entering- the rate of oxygen exiting- the rate of oxygen used equally to the rate of accumulation of oxygen in the system (Clarke 2013). This was done to test the effect of OTR on β-Carotene production. The results indicated that the concentration of β-carotene (704.1 mg/l) was the highest in a culture grown at a maximum OTR of 20.5 mmol/(l h) (Mantzouridou et al. 2005).

Another engineering strategy used by Mantzouridou et al. (2017) to produce lycopene is to optimize the fermentation with lycopene cyclase inhibitor. The addition of this inhibitor involves a lower production of the carotenoid β-and derived, and consequently a major production of the carotenoid Ψ-end derived. Instead, an approach used to increase β-carotene production in E. coli consists in the engineering of the methylerythritol phosphate (MEP) pathway to improve the production of IPP (The inositol pyrophosphate) and DMAPP (Dimethylallyl Diphosphate). Both are precursors of β-carotene then improving their synthesis, we will have a major production of β-carotene. Moreover, the engineering of the TCA pathway is used as a strategy to refine the production of β-carotene because, in this way, they increase the production of ATP and NADH, so it will be easier to produce the chemical (Zhao et al. 2013). We know that Acetyl-coA is a precursor of carotenoid synthesis. So if we wanted to increase the production of lycopene or β-carotene, in theory, we should intensify the concentration of Acetyl-coA. Chen et al. (2016) have worked on this through a process of host engineering in which YPL062W, a distant genetic locus in S. cerevisiae CEN.PK2, was deleted. In this way, little acetate was accumulated, and an approximately 100 % increase in cytosolic acetyl-CoA pool was achieved relative to that in the parental strain (Chen et al. 2016).

Larroude et al. (2018) used an interesting strategy to improve the production of β-carotene. Their strategy is slightly dissimilar from the others because they developed a combinatorial synthetic biology approach based on Golden Gate DNA assembly to screen the optimum promoter-gene pairs for each transcriptional unit expressed. The major conclusion of their results is that the cassette with the three genes controlled by TEFp

is the optimum producer. So, they constructed a new car-cassette, where the three genes (GGS1, carB, and carPR) are under the control of the TEF1 promoter (Larroude et al. 2018). Therefore, we looked at some of the strategies that have been used in different studies in order to give a broader and more complete view of the topic and to make it clear how synthetic biology can offer countless possibilities in the field of research.

Table B. In this table is shown the results derived from some research in which we can see that the strategies adopted are different and various. We can see as strategies, for example, control of oxygen transfer rate, optimization of fermentation with lycopene cyclase inhibitor, Regulation of lycopene synthesis pathway expression, Engineering MEP pathway for IPP and DMAPP supply and central pathway (TCA, PPP) for carbon flux, increase of acetyl-CoA pool and optimization of the lycopene synthesis pathway

**Host strain**	**Descriptions**	**Products and titers**	**Engineering strategies**	**References**
Blakeslea trispora	Native producer of carotenoids	β-Carotene 704.1 mg/L	Control of oxygen transfer rate	Mantzouridou et al. 2005
Blakeslea trispora	Native producer of carotenoids	Lycopene, 256 mg/L	Optimization of fermentation with lycopene cyclase inhibitor	Mantzouridou et al. 2017
Escherichia coli	Genetically tractable, non-native producer	Lycopene, 0.5 g/g DCW	Regulation of lycopene synthesis pathway expression	Coussement et al. 2017
Escherichia coli	Genetically tractable, non-native producer	β-Carotene, 2.1 g/L	Engineering MEP pathway for IPP and DMAPP supply and central pathway (TCA, PPP) for carbon flux	Zhao et al. 2013
Saccharomyces cerevisiae	Genetically tractable, non-native producer	Lycopene, 56 mg/g DCW	Increase of acetyl-CoA pool and optimization of lycopene synthesis pathway via genome manipulation	Chen et al. 2016

Corynebacterium glutamicum	Native producer of C50 Carotenoid	β-Carotene, 7 mg/L	Deletion of *crtR* and integration of *crt* pathway genes	Henke et al. 2018
Rhodobacter sphaeroides	Phototroph with carotenogenic genes	Lycopene, 10 mg/g DCW	Replacement of *crtI*, augmentation of MEP pathway, and block of PPP pathway	Su et al. 2018
Yarrowia lipolytica	Genetically tractable, non-native producer	β-Carotene, 6.5 g/L	Optimization of promoter-gene pairs of heterologous *crt* pathway	Larroude et al. 2018
Yarrowia lipolytica	Genetically tractable, non-native producer	β-Carotene, 4 g/L	Iterative integration of multiple-copy pathway genes	Gao et al. 2017
Yarrowia lipolytica	Genetically tractable, non-native producer	β-Carotene 0.22636 $[mmol \cdot gDW^{-1} \cdot h^{-1}[mmol \cdot gDW^{-1} \cdot h^{-1}]]$	Iterative integration of three genes (GGS1, carPR, carP)	**Our work** (strain with 3 Knockouts)
Yarrowia lipolytica	Genetically tractable, non-native producer	β-Carotene 0.22634 $[mmol \cdot gDW^{-1} \cdot h^{-1}[mmol \cdot gDW^{-1} \cdot h^{-1}]]$	Iterative integration of three genes (GGS1, carPR, carP)	**Our work** (strain with 2 Knockouts)
Yarrowia lipolytica	Genetically tractable, non-native producer	β-Carotene 0.22635 $[mmol \cdot gDW^{-1} \cdot h^{-1}[mmol \cdot gDW^{-1} \cdot h^{-1}]]$	Iterative integration of three genes (GGS1, carPR, carP)	**Our work** (strain with 4 Knockouts)

C. Comparison of algorithms and methods for succinate production

Table C shows a comparison between our research work and other algorithms mentioned, where the objective is to maximize the production of succinate. From left to right, we reported: Approach/ Algorithm, year of publication, organism, main carbon source, medium, Genetic modification, Results, and References.

Table C. In this table, we have selected the best strains (in terms of succinate production), comparing our work with some articles in the literature. As we can see, each strain has a variable number of knockout genes. In the table from left to right, we reported: Approach/ Algorithm, year of publication, organism, main carbon source, medium, Genetic modification, Results, and References. In this table, only studies that use computational methods to guide the corresponding experimental engineering strategy are considered. Even though FBA and TFBA are not strained design algorithms as such and, consequently, do not allow the direct identification of cellular targets, they have been widely used as a valuable tool in rational strain design. FBA flux balance analysis, CDM Chemically defined medium, TFBA thermodynamics-based flux balance analysis.

**Approach/ Algorithm**	**Organism**	**Main Carbon Source**	**Medium**	**Genetic Modification**	**Result**	**References**
FBA	*E. coli*	Sorbitol	Complex + anaerob	*DldhA, Dpfl + sfcA overexpression*	38% improvement in succinate productivity	Lee et al., 2002
FBA	*E. coli*	Glucose	Complex + anaerob	*DptsG, DpykF and DpykA*	235% increase in titer	Lee et al., 2005
FBA	*S. cerevisiae*	Glucose	aerobic glucose-limited conditions	SDH-complex, ZWF1, PDC6, U133, U221	0.39 mg/(g glucose x h)	Patil et al., 2005

FBA	*E. coli*	Glucose	Complex + anaerob	*DptsG, DiclR + pyc overexpression*	760% increase in succinate yield	Wang et al., 2006
FBA	*S. cerevisiae*	Glucose	Not available	*sdh3, ser3, ser33*	0.90 g of succinate/L	Otero et al., 2013
TFBA	*E. coli*	Glucose	Complex + anaerob	*DldhA, Dpflb, DptsG, Dppc + PEPCK from Actinobacillus succinogenes*	60% improvement in succinate titer	Singh et al., 2011
FBA	*S. cerevisiae*	Glucose	Minimal supplemented with vitamins and amino acids + anaerob	*Ddic1*	0.02 (C-mol/C-mol) yield	Agren et al., 2013
OptGene	*S. cerevisiae*	Glucose	Minimal supplemented with vitamins + aerob	*Dsdh3, Dser3, Dser33 + icl1 overexpression*	30-fold improvement in succinate titer	Otero et al., 2013
CASOP	*E. coli*	Glucose	Complex + aerob	*DsdhA, DackA-pta, DpoxB, DmgsA, DiclR + pyc overexpression*	52% improvement in specific productivity and 58% in yield	Yang et al., 2014

FBA	*E. coli*	Glucose	Minimal + anaerob	*DackA-pta + pgl, tktA, talB, sthA, dcuB, dcuC overexpression + pepck from Actinobacillus succinogenes + pyc from C. glutamicum + mutated zwf243 and gnd361 from Corynebacterium glutamicum*	52% improvement in yield	Meng et al. 2016
FBA	*M. succinipro ducens*	Glucose	Complex + anaerob	*DackA-pta and DldhA*	35% improvement in maximum productivity	Choi et al., 2016
FBA	*M. succinipro ducens*	Sucrose + Glycerol	CDM + anaerob	*DackA-pta and DldhA*	34% improvement in overall productivity and 21% improvement in yield	Lee et al., 2016
FBA	*S. cerevisiae*	Glucose	Rich medium	***GLT1****(IV), DCD1[XII], ADO1(X), PNP1[XII], LAT1[XIV], PSD1[XIV], ALE1(XV),****ALD6****[XVI] , GPH1[XVI]*	3.1215 $[mmol \cdot gDW^{-1} \cdot h^{-1}]$ Max Productivity: 0.14841 $[h^{-1}]$ Max Yield: 0.2282	**Our work** (strain with maximal Succinate Production)

FBA	*S. cerevisiae*	Glucose	Rich Medium	*PDB1(II), GLT1(IV),ALD6[XVI] , GPH1[XVI]*	3.1027 $[mmol \cdot gDW^{-1} \cdot h^{-1}]$ Max Productivity: 0.14795 $[h^{-1}]$ Max Yield: 0.2295	**Our work** (strain with a minimal number of gene Knockouts)
FBA	*S. cerevisiae*	Glucose	Rich medium	*GLT1(IV), ALD6 (XVI), GPH1 (XVI), YPR127W*	2,9562 [$mmol \cdot gDW^{-1} \cdot h^{-1}]$ Max Productivity: 0.14064 $[h^{-1}]$ Max Yield: 0.2395	**Our work** (strain with maximal yield)

D. Analysis of the role of genes of S. cerevisiae

We present an additional analysis to determine the role of frequently silenced genes, GLT1, ALD6, and GPH1 in S. cerevisiae by our multiobjective evolutionary algorithm framework. In this additional analysis, genes were first switched off individually, then switched off pair-wise, and lastly switched off altogether to understand the impact that silencing these genes has on the microorganism. These analyses were performed using MATLAB and then represented as graphs produced by Escher (King et al., 2015).

We notice a higher flow leading to an increase of the isocitrate concentration in the mitochondrial compartment (isocitrate is a key organic acid of the TCA cycle) when the ALD6 gene was knocked out (see Figure D1). The reaction involving the *citrate transport* in Figure D1 that catalyzes the transport of isocitrate from mitochondria compartment to cytoplasm shows the value of this flux is lower in the strain (where ALD6 is knocked out) than the wild type. In fact, next to the name of the enzyme that catalyzes the reaction, there are three values, the first represents the flux of the strain obtained by silencing of ALD6, the second represents a wild type, and the third represents the difference between these two values (more reddish the edges are higher the difference and more bluish the edges are lower this difference in Figure D1). The difference is equal to 2.28, which means that in the strain, there is a major concentration of isocitrate in the mitochondria. Moreover, there is a lower activity of *isocitrate dehydrogenase* (NAD+) in the strain than in the wild type. This enzyme catalyzes the conversion of isocitrate in 2-oxoglutarate. It is known that the isocitrate can be transformed into succinate by *isocitrate lyase*. Therefore, by switching off the ALD6 gene, there is a higher isocitrate concentration given by a lower activity of the enzyme *citrate transport* and *isocitrate dehydrogenase* (NAD+) (Figure D1). This could lead to increased production of succinic acid. Hence, looking at the TCA cycle, the flows are channeled towards a higher accumulation of isocitrate at the mitochondrion level. Similarly, in the case of the strain obtained by silencing the GLT1 gene, we find a redirection of the fluxes leads to a higher accumulation of isocitrate at the mitochondrial level, although it was less than the strain in which the ALD6 gene was knocked out (Figure D2).

Figure D3 presents the analyses for silencing the GPH1 gene. This analysis shows that the flow of ATP: D-glucose 6-phosphotransferase is increased highly. This enzyme catalyzes the conversion reaction of D-glucose to D-glucose 6- phosphate. This results in much higher production of D-glucose 6-phosphate cytoplasmic and consequently increased use of glucose. Therefore, this could lead to a higher yield of succinate. We observed that in the case of the strain obtained by knocking out of the GPH1 gene, the flux of isocitrate lyase reaction (that catalyzes the conversion of isocitrate into succinate) increases from zero in the wild type to 0.604 in the strain. An increase in this flux value suggests that the knockout of the GPH1 gene increases the synthesis of the enzyme isocitrate lyase and therefore increases the production of succinate in the microorganism. The increase in the flux values when the GPH1 gene was more prominent than knocking out GLT1 and ALD6

confirms the significance of GPH1 knockout. We can conclusively demonstrate that knocking genes ALD6 and GLT1 genes increase the concentration of isocitrate in the mitochondrial compartments, leading to higher succinate production. Additionally, knocking out GPH1 genes help the growth of Isocitrate lyase, which converts isocitrate into succinate, leading to higher succinate production. These analyses also validate the finding of our multiobjective evolutionary algorithms optimization of strains for automatizing metabolic engineering.

In our final analysis, we first combined the knockout of two genes one at a time to perform four different analyses and finally turned off all three genes simultaneously. We observed that the metabolism of the strain obtained by knocking out all three genes together includes the characteristics of the three strains obtained by knocking out each gene separately (see Figures D1, D2, and D3). This means there is a higher accumulation of isocitrate in the mitochondrion, D-glucose 6-phosphate cytoplasm, and especially the activation of the isocitrate lyase enzyme.

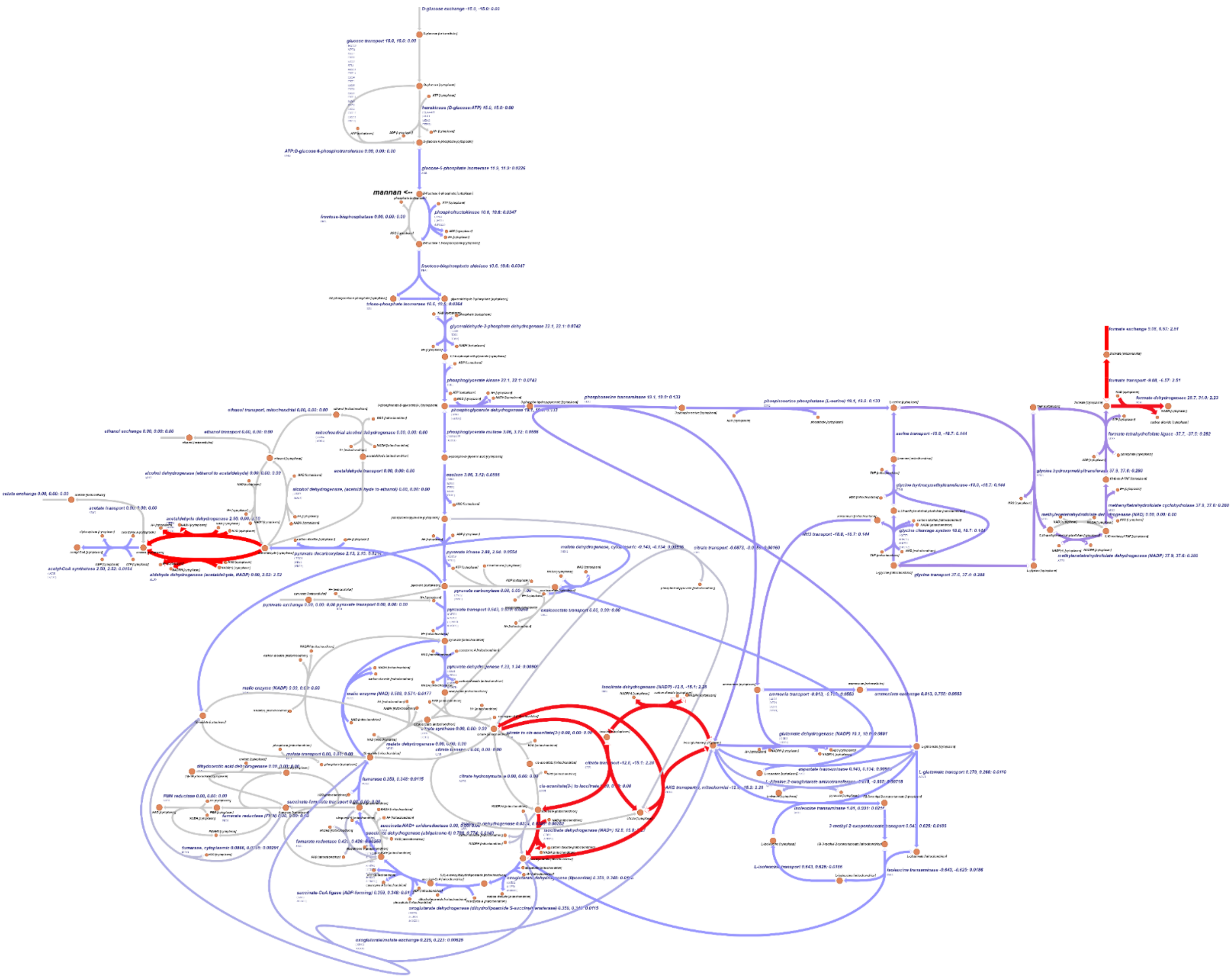

Figure D1. Confront of the Krebs cycle of S. cerevisiae wild type and the strain with ALD6 gene KO. The pathways are shown in blue and red colors, and some pathways are not colored. These colors represent the difference between wild-type flux and the flux of the strain obtained by silencing the ALD6 gene. The pathways colored in red represent a higher difference of value than the pathways colored in blue. The zoom-in versions of Figure D1 are shown in Figure D1-A, D1-B, and D1-C.

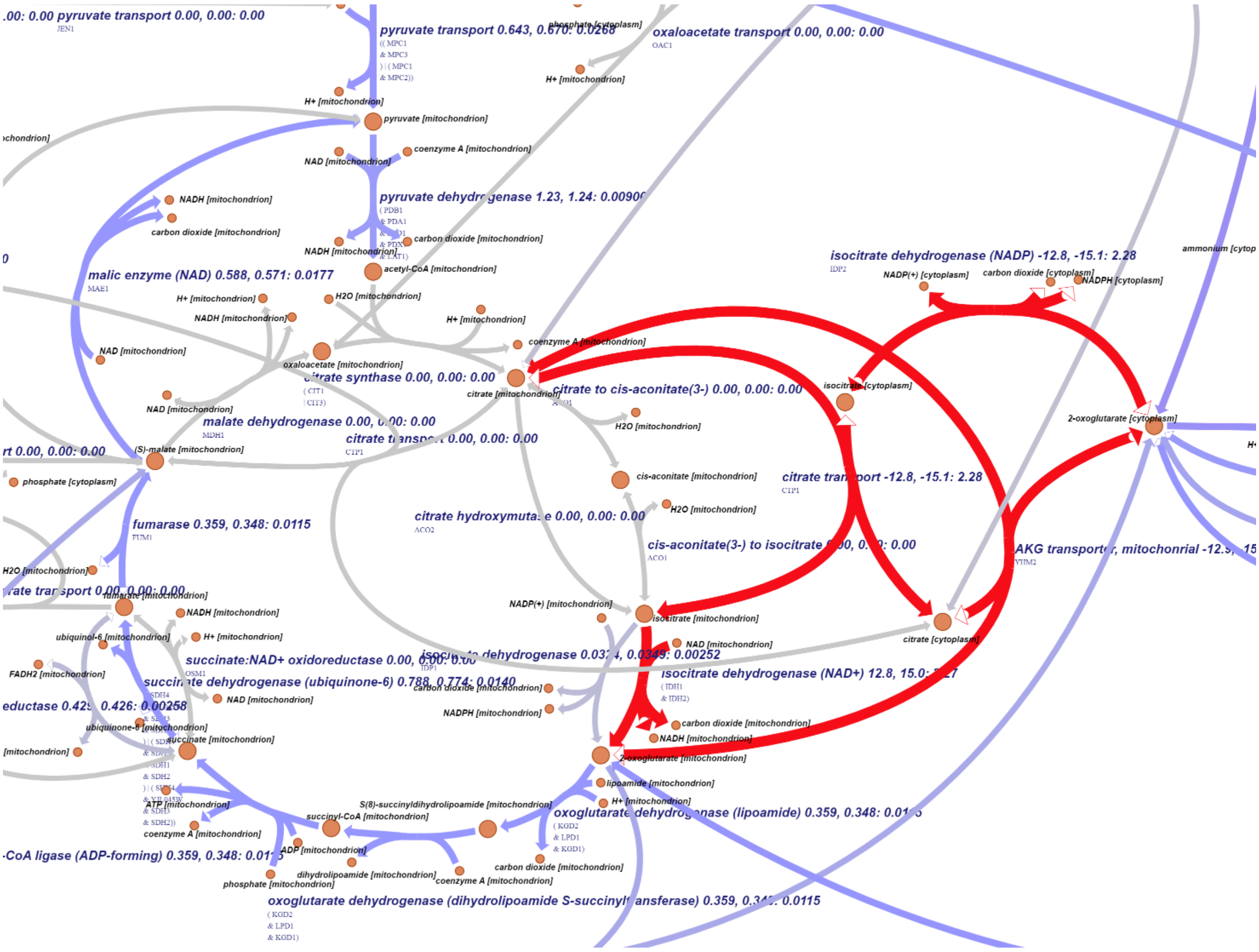

Figure D1-A. Flux changes at the levels of the tricarboxylic acid cycle part of Figure D1. This part focuses on the fluxes that are affected by the Knockout of the ALD6 gene. In red we have highlighted the fluxes that are mainly changed by the Knockout of ALD6. One of these enzymes is the citrate transport, which catalyzes the transport of isocitrate from the mitochondrial compartment to the cytoplasm. The value of the flux of this reaction is lower in the strain (where ALD6 is knocked out) than in the wild type. Therefore, through the Knockout of the gene ALD6, we obtained a major concentration of isocitrate in the mitochondrial compartment.

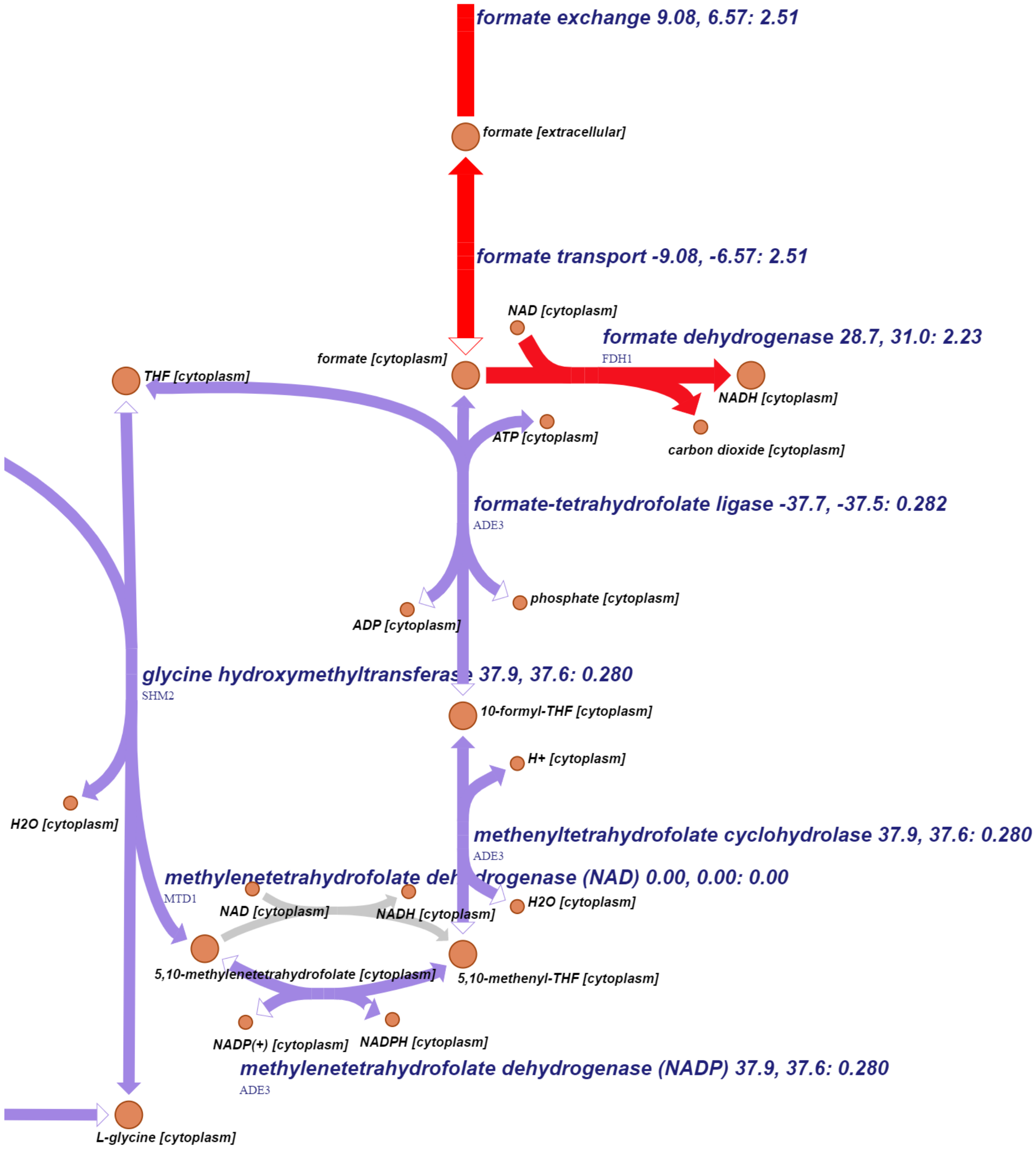

Figure D1-B. This part of Figure D1 shows the change that derives from the Knockout of the ALD6 gene lies in the variation of the formate concentration. Formate is a monocarboxylic acid anion that is the conjugate base of formic acid. It has a role as a metabolite in the S. cerevisiae metabolism. This part shows that knocking out the ALD6 gene has a minor activity of enzyme ‘formate dehydrogenase’. This implies a major concentration of formate in the cytoplasm and consequently in a minor production of NADH.

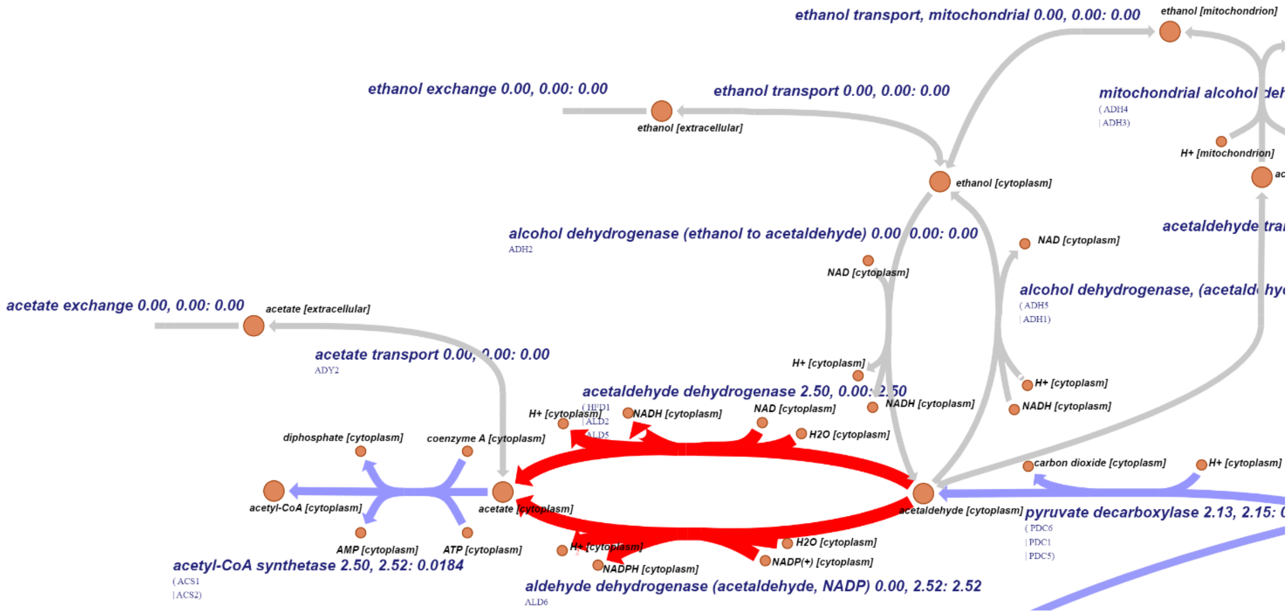

Figure D1-C. This part of Figure D1 shows the effect of the knockout of the ALD6 gene that leads to the activation of a specific isoenzyme and simultaneously to the inactivation of the other corresponding enzyme. This part indicates that the enzyme ‘aldehyde dehydrogenase (acetaldehyde, NADP)’ in the wild type is fully active, while the enzyme ‘acetaldehyde dehydrogenase’ has a flow of zero. Knocking out the ALD6 gene has a contrary situation, that is, the enzyme ‘acetaldehyde dehydrogenase’ increases its flow, while the enzyme ‘aldehyde dehydrogenase (acetaldehyde, NADP)’ resets it.

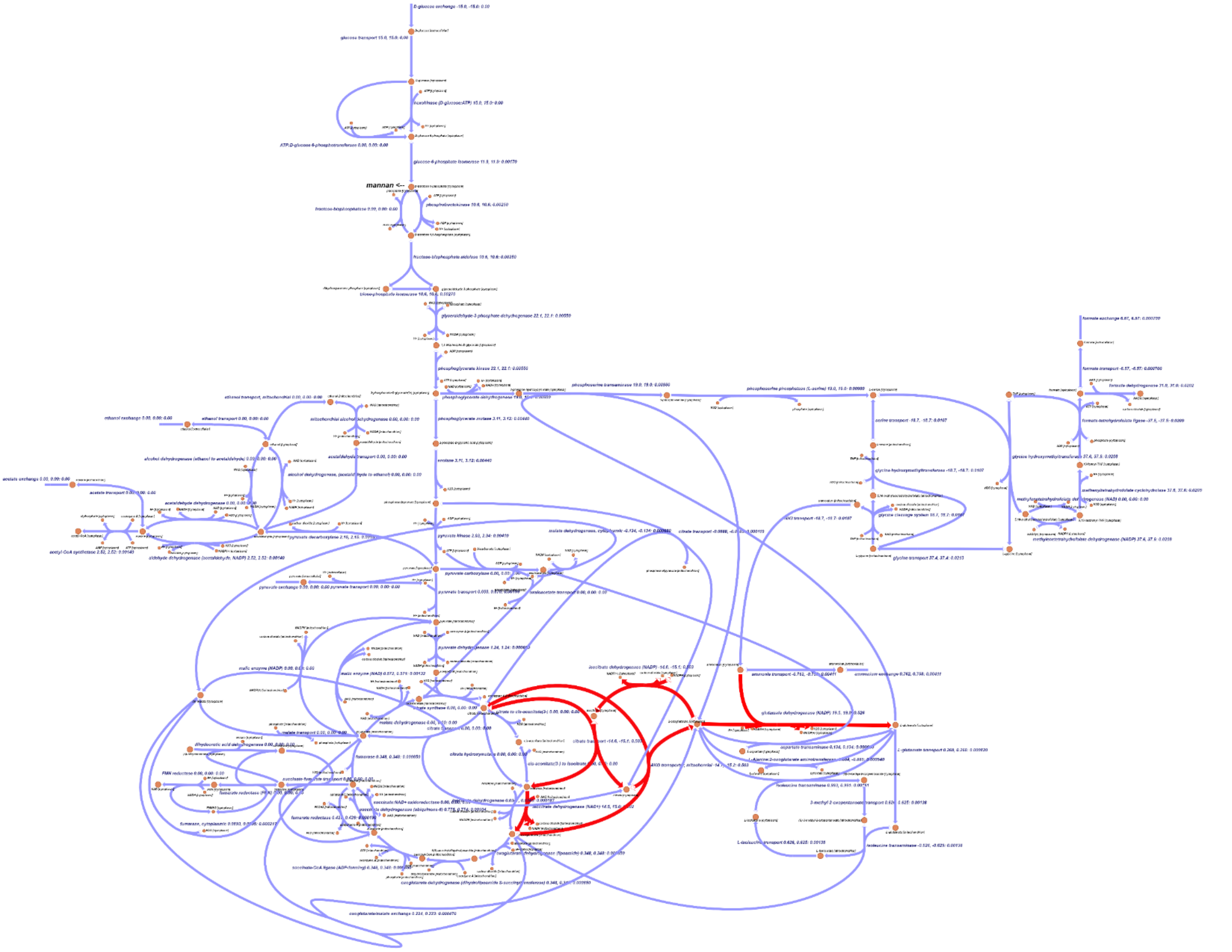

Figure D2. Confront of Krebs cycle of S. cerevisiae wild type and the strain with GLT1 gene knockout. Red and blue colors represent the difference between wild-type flux and the flux of the strain obtained by silencing the GLT1 gene. The pathways that are colored in red represent a higher difference of value than pathways that are colored in blue.

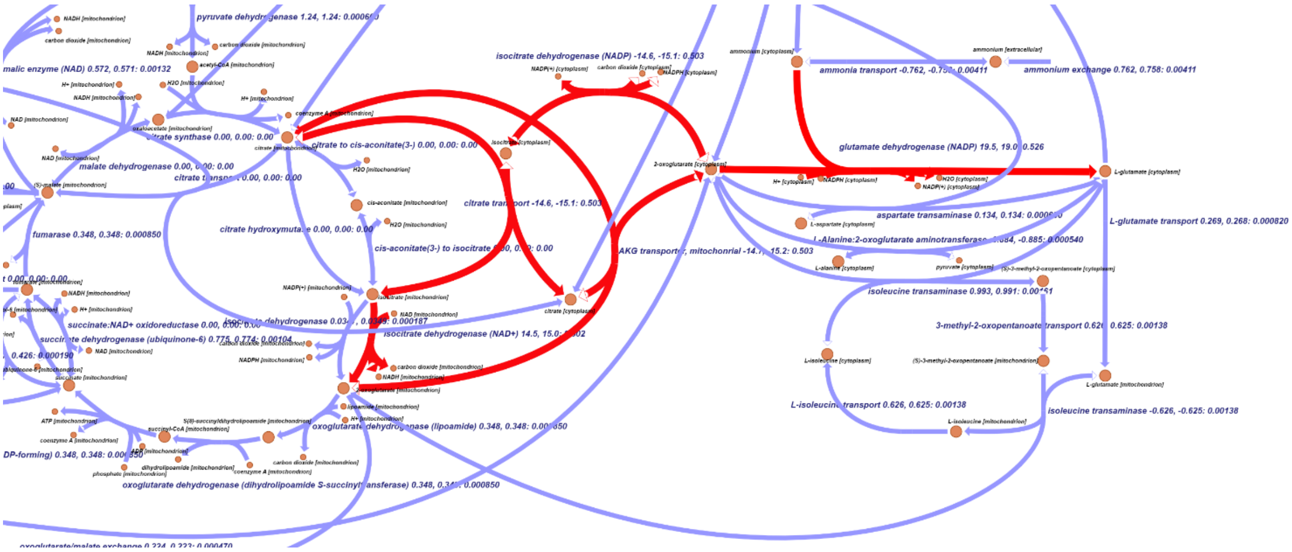

Figure D2-A. Similar to Figure D1-A, in the case of the strain obtained by silencing the GLT1 gene, we find a redirection of the fluxes leads to a higher accumulation of isocitrate at the mitochondrial level, although it was less than the strain in which the ALD6 gene was knocked out (Figure D1-A).

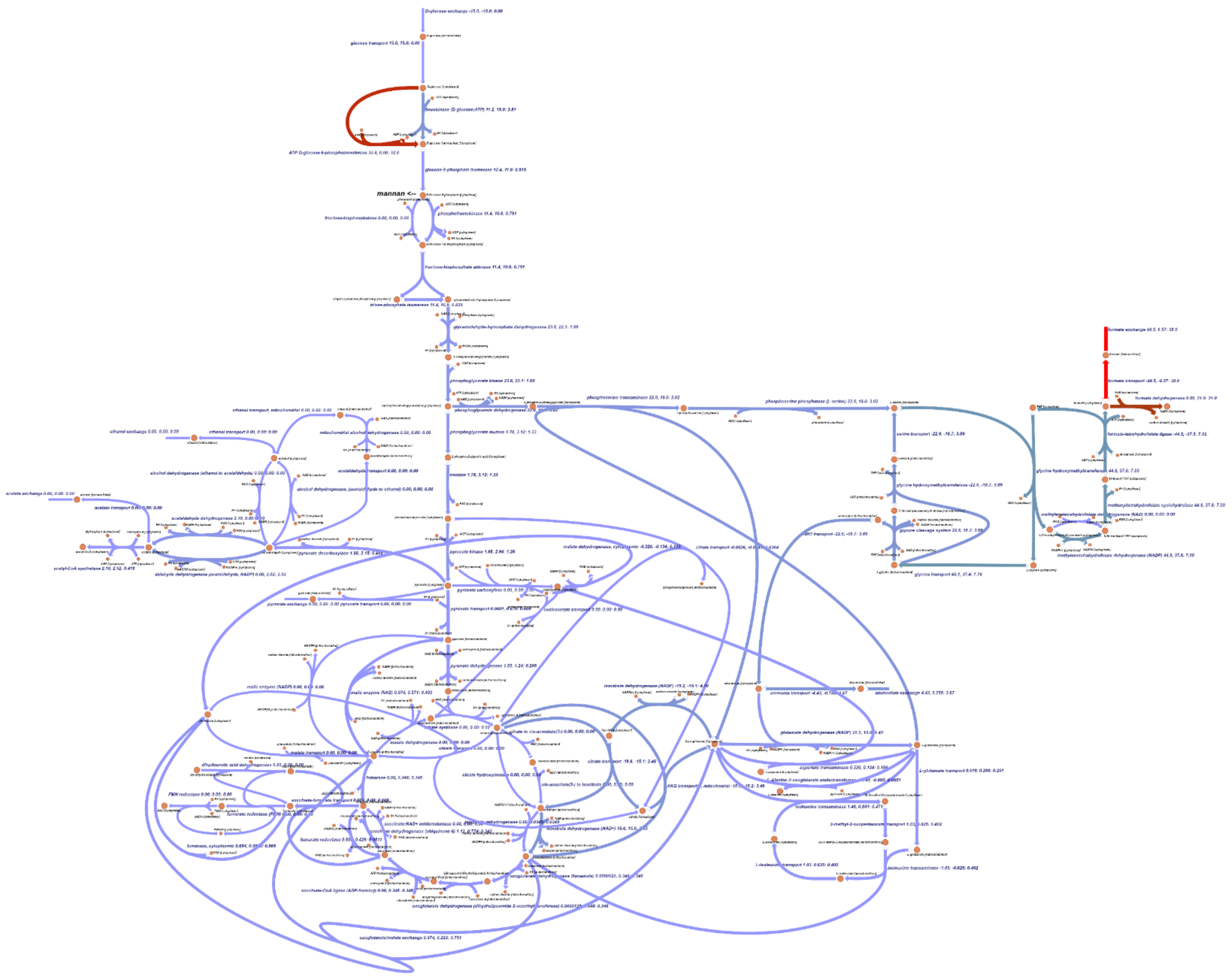

Figure D3. Confront of Krebs cycle of S. cerevisiae wild type and the strain with GPH1 gene knockout. Red and blue colors represent the difference between wild-type flux and the flux of the strain obtained by silencing the GPH1 gene. The pathways that are colored in red represent a higher difference of value than pathways that are colored in blue.

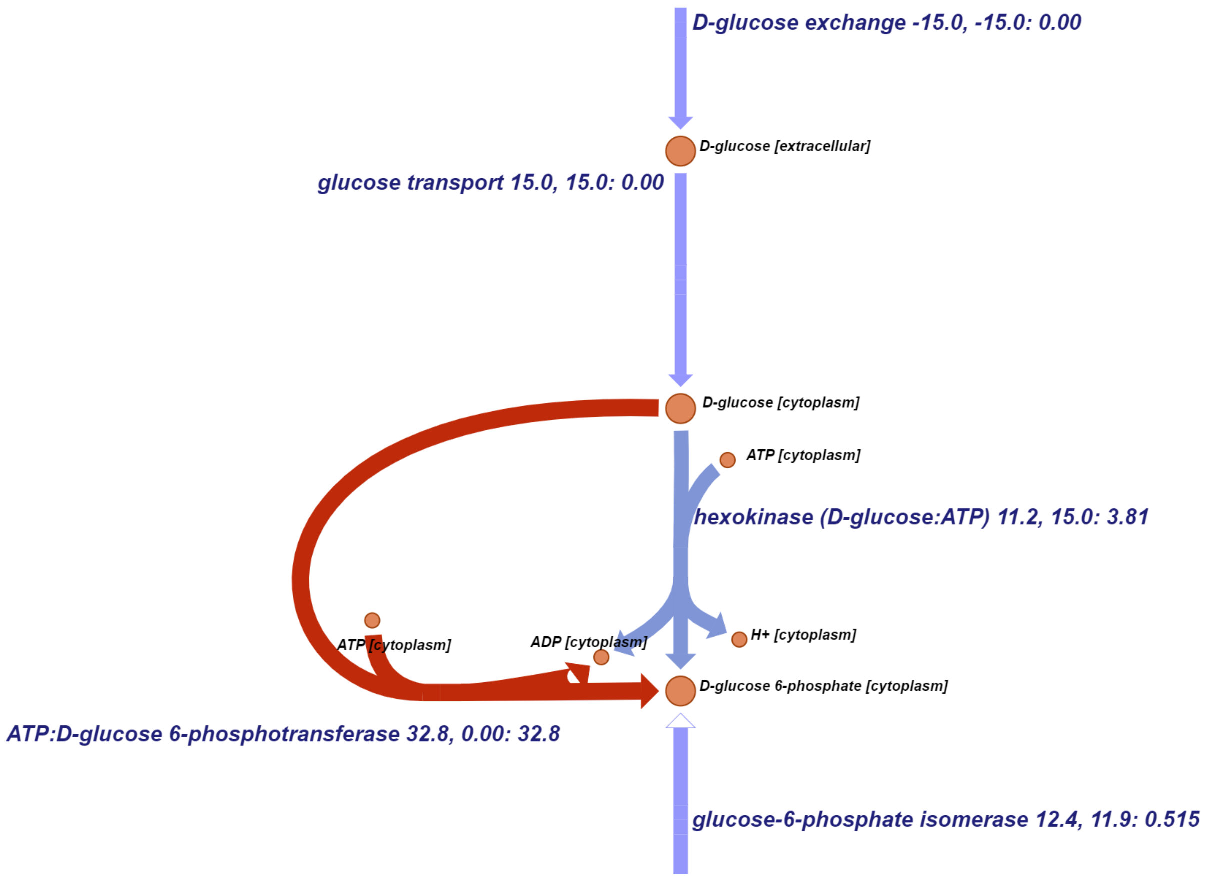

Figure D3-A. This part of Figure D3 shows that the flow of ATP: D-glucose 6-phosphotransferase is increased highly. This enzyme catalyzes the conversion reaction of D-glucose to D-glucose 6- phosphate. This results in much higher production of D-glucose 6-phosphate cytoplasm and consequently increased the use of glucose. Therefore, this may have led to a higher yield of succinate. We also notice a substantial increase in this flux, in fact, it passed from 0.00 in the wild type to 32.8 in the strain with the GPH1 gene KO.

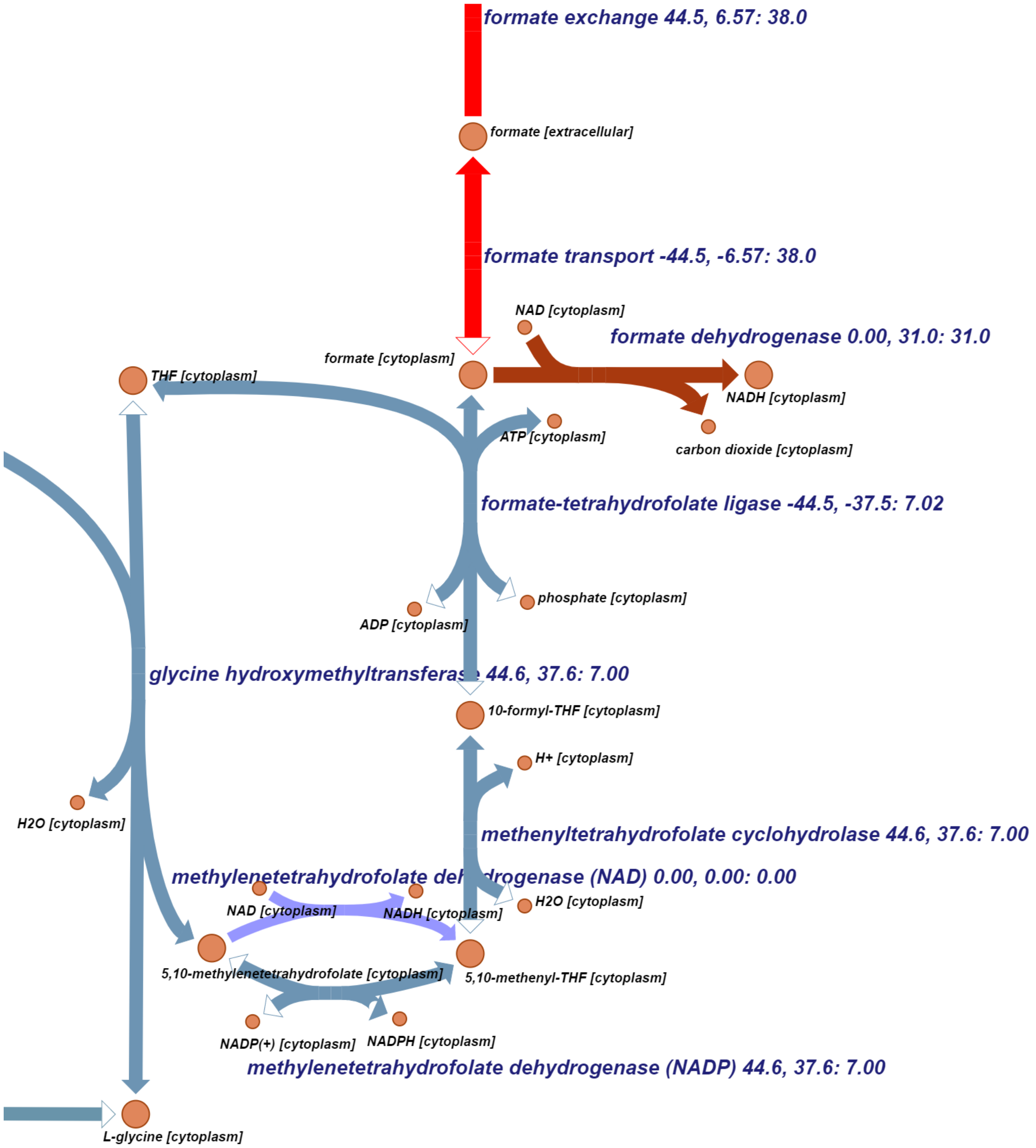

Figure D3-B. This part of Figure D3 shows that knocking out of the GPH1 gene brings the 'formate dehydrogenase' reaction flow to zero. The GPH1 gene affects this reaction more than the ALD6 gene. In fact, as per Figure D1-A, where the knockout of the ALD6 gene led to a decrease in the flow of 'formate dehydrogenase,' in the case of knocking out the GPH1 gene the similar flow is brought to zero. This will produce a much larger increase in formate concentration, compared to a noticeable decrease in cytoplasmic NADH concentration.

E. List of Abbreviations

Ac-ac- CoA	Acetyl- acetyl coA
ACDH	Activated acetaldehyde dehydrogenase
ACS	acetyl-CoA synthetase
AGP3	glutamate permease
ATP	Adenosine triphosphate
BPCY	Biomass Product Coupled Yield
carB	phytoene dehydrogenase
carPR	phytoene synthase/ lycopene cyclase
CNM	Central Nitrogen Metabolism
CoA	Coenzyme A
FAD(H2)	Dihydroxyflavone-adenine dinucleotide
FBA	Flux Balance Analysis
FPP	Farnesyl diphosphate
FVA	Flux Variability Analysis
GGS1	geranylgeranyl diphosphate synthase
GOGAT	Glutamate synthase
GPR	Gene- Protein- Reaction
HMG-coA	3- Hydroxy- 3- methylglutaryl coenzyme A
HMGS	Hydroxymethylglutaryl- CoA- synthase
KO	Knockout
MOEA	Multiobjective Evolutionary Algorithm
POME	Pareto Optimal Metabolic Engineering algorithm
MVA	Mevalonate
NADH	Nicotinamide adenine dinucleotide
NADPH	Nicotinamide adenine dinucleotide phosphate
PABA	para-amino benzoate
PDC	Pyruvate decarboxylase
PDC6	pyruvate decarboxylase
PDH	Pyruvate dehydrogenase
pFBA	parsimonious Flux Balance Analysis
SDH- complex	Succinate dehydrogenase complex
TCA	Tricarboxylic Acid
THR1	homoserine kinase
YNB	Yeast Nitrogen Base medium
YPD	Yeast Extract- Peptone- Dextrose medium
WT	Wild Type strain
sgRNA	Single strain RNA

References

Agren, R. , Otero, J. M. , & Nielsen, J. (2013). Genome-scale modeling enables metabolic engineering of *Saccharomyces cerevisiae* for succinic acid production. *Journal of Industrial Microbiology and Biotechnology*, *40*(7), 735−747.

Chen, Y. , Xiao, W. , Wang, Y. , Liu, H. , Li, X. , & Yuan, Y. (2016). Lycopene overproduction in *Saccharomyces cerevisiae* through combining pathway engineering with host engineering. *Microbial Cell Factories*, *15*, 113.

Choi, S. , Song, H. , Lim, S. W. , Kim, T. Y. , Ahn, J. H. , Lee, J. W. , Lee, M. H. , & Lee, S. Y. (2016). Highly selective production of succinic acid by metabolically engineered *Mannheimia succiniciproducens* and its efficient purification. *Biotechnology and Bioengineering*, *113*(10), 2168−2177.

Ciccazzo, A. , Conca, P. , & Nicosia, G. , et al (2008). An advanced clonal selection algorithm with ad-hoc network-based hypermutation operators for synthesis of topology and sizing of analog electrical circuits. In *Seventh International Conference on artificial immune systems—ICARIS, 10th–13th August 2008*. LNCS (Vol. 5132, pp. 60−70). Springer.

Clarke, K. G. (2013). The oxygen transfer rate and overall volumetric oxygen transfer coefficient. *Bioprocess Engineering*, 147−170.

Coussement, P. , Bauwens, D. , Maertens, J. , & De Mey, M. (2017). Direct combinatorial pathway optimization. *ACS Synthetic Biology*, *6*, 224−232.

Deb, K. (2001). *Multiobjective optimization using evolutionary algorithms*. Wiley.

Gao, S. , Tong, Y. , Zhu, L. , Ge, M. , Zhang, Y. , Chen, D. , Jiang, Y. , & Yang, S. (2017). Iterative integration of multiple-copy pathway genes in *Yarrowia lipolytica* for heterologous β-carotene production. *Metabolic Engineering*, *41*, 192−201.

Henke, N. A. , Wiebe, D. , Pérez-García, F. , Peters-Wendisch, P. , & Wendisch, V. F. (2018). Coproduction of cell-bound and secreted value-added compounds: Simultaneous production of carotenoids and amino acids by *Corynebacterium glutamicum* . *Bioresource Technology*, *247*, 744−752.

King, Z. A. , Dräger, A. , Ebrahim, A. , Sonnenschein, N. , Lewis, N. E. , & Palsson, B. O. (2015). Escher: A web application for building, sharing, and embedding data-rich visualizations of biological pathways. *PLoS Computational Biology*, *11*(8):e1004321.

Larroude, M. , Celinska, E. , Back, A. , Thomas, S. , Nicaud, J. M. , & Ledesma-Amaro, R. (2018). A synthetic biology approach to transform *Yarrowia lipolytica* into a competitive biotechnological producer of β-carotene. *Biotechnology and Bioengineering*, *115*(2), 464−472.

Lee, J. W. , Yi, J. , Kim, T. Y. , Choi, S. , Ahn, J. H. , Song, H. , Lee, M. H. , & Lee, S. Y. (2016). Homo-succinic acid production by metabolically engineered *Mannheimia succiniciproducens* . *Metabolic Engineering*, *38*, 409−417.

Lee, S. J. , Lee, D. Y. , Kim, T. Y. , Kim, B. H. , Lee, J. , & Lee, S. Y. (2005). Metabolic engineering of *Escherichia coli* for enhanced production of succinic acid, based on genome comparison and in silico gene knockout simulation. *Applied and Environmental Microbiology*, *71*(12), 7880−7887.

Lee, S. Y. , Hong, S. H. , & Moon, S. Y. (2002). In silico metabolic pathway analysis and design: Succinic acid production by metabolically engineered *Escherichia coli* as an example. *Genome Inf. 13*, 214–223.

Mantzouridou, F. T. , & Naziri, E. (2017). Scale translation from shaken to diffused bubble aerated systems for lycopene production by *Blakeslea trispora* under stimulated conditions. *Applied Microbiology and Biotechnology*, *101*, 1845–1856.

Mantzouridou, F. , Roukas, T. , & Achatz, B. (2005). Effect of oxygen rate on β-carotene production from synthetic medium by *Blakeslea trispora* in shake flask culture. *Enzyme and Microbial Technology*, *37*, 687–694.

Meng, J. , Wang, B. , Liu, D. , Chen, T. , Wang, Z. , & Zhao, X. (2016). High-yield anaerobic succinate production by strategically regulating multiple metabolic pathways based on stoichiometric maximum in *Escherichia coli* . *Microbial Cell Factories*, *15*, 141.

Otero, J. M. , Cimini, D. , Patil, K. R. , Poulsen, S. G. , Olsson, L. , & Nielsen, J. (2013). Industrial systems biology of *Saccharomyces cerevisiae* enables novel succinic acid cell factory. *PLoS One*, *8*(1), e54144.

Patané, A. , Jansen, G. , Conca, P. , Carapezza, G. , Costanza, J. , & Nicosia, G. (2019). Multiobjective optimization of genome-scale metabolic models: The case of ethanol production. *Annals of Operations Research*, *276*(1), 211–227

Patil, K. R. , Rocha, I. , Förster, J. , & Nielsen, J. (2005). Evolutionary programming as a platform for in silico metabolic engineering. *BMC Bioinformatics*, *6*(1), 1–12.

Singh, A. , Cher Soh, K. , Hatzimanikatis, V. , & Gill, R. T. (2011). Manipulating redox and ATP balancing for improved production of succinate in *E. coli* . *Metabolic Engineering*, *13*(1), 76–81.

Su, A. , Chi, S. , Li, Y. , Tan, S. , Qiang, S. , Chen, Z. , & Meng, Y. (2018). Metabolic redesign of *Rhodobacter sphaeroides* for lycopene production. *Journal of Agricultural and Food Chemistry*, *66*, 5879–5885.

Valenzuela, L. , Guzmán-León, S. , Coria, R. , Ramírez, J. , Aranda, C. , & González, A. (1995). A NADP-glutamate dehydrogenase mutant of the petit-negative yeast *Kluyveromyces lactis* uses the glutamine synthetase-glutamate synthase pathway for glutamate biosynthesis. *Microbiology*, *141*(10), 2443–2447.

Wang, Q. , Chen, X. , Yang, Y. , & Zhao, X. (2006). Genome-scale in silico aided metabolic analysis and flux comparisons of *Escherichia coli* to improve succinate production. *Applied Microbiology and Biotechnology*, *73*(4), 887–894.

Yang, J. , Wang, Z. , Zhu, N. , Wang, B. , Chen, T. , & Zhao, X. (2014). Metabolic engineering of *Escherichia coli* and in silico com- paring of carboxylation pathways for high succinate productivity under aerobic conditions. *Microbiological Research*, *169*(5–6), 432–440.

Zhao, J. , Li, Q. , Sun, T. , Zhu, X. , Xu, H. , Tang, J. , Zhang, X. , & Ma, Y. (2013). Engineering central metabolic modules of *Escherichia coli* for improving b-carotene production. *Metabolic Engineering*, *17*, 42–50.
